# Multimodal human thymic profiling reveals trajectories and cellular milieu for T agonist selection

**DOI:** 10.3389/fimmu.2022.1092028

**Published:** 2023-01-20

**Authors:** Marte Heimli, Siri Tennebø Flåm, Hanne Sagsveen Hjorthaug, Don Trinh, Michael Frisk, Karl-Andreas Dumont, Teodora Ribarska, Xavier Tekpli, Mario Saare, Benedicte Alexandra Lie

**Affiliations:** ^1^ Department of Medical Genetics, Oslo University Hospital, University of Oslo, Oslo, Norway; ^2^ Department of Pathology, Oslo University Hospital, Oslo, Norway; ^3^ Institute for Experimental Medical Research, Oslo University Hospital, University of Oslo, Oslo, Norway; ^4^ KG Jebsen Centre for Cardiac Research, University of Oslo, Oslo, Norway; ^5^ Department of Cardiothoracic Surgery, Oslo University Hospital, Oslo, Norway

**Keywords:** human thymus, autoimmunity, T cell development, T agonist selection, antigen-presenting cells, single-cell RNA sequencing, spatial transcriptomics, multi-modal

## Abstract

To prevent autoimmunity, thymocytes expressing self-reactive T cell receptors (TCRs) are negatively selected, however, divergence into tolerogenic, agonist selected lineages represent an alternative fate. As thymocyte development, selection, and lineage choices are dependent on spatial context and cell-to-cell interactions, we have performed Cellular Indexing of Transcriptomes and Epitopes by sequencing (CITE-seq) and spatial transcriptomics on paediatric human thymu​​s. Thymocytes expressing markers of strong TCR signalling diverged from the conventional developmental trajectory prior to CD4^+^ or CD8^+^ lineage commitment, while markers of different agonist selected T cell populations (CD8αα(I), CD8αα(II), T_(agonist)_, T_reg_(diff), and T_reg_) exhibited variable timing of induction. Expression profiles of chemokines and co-stimulatory molecules, together with spatial localisation, supported that dendritic cells, B cells, and stromal cells contribute to agonist selection, with different subsets influencing thymocytes at specific developmental stages within distinct spatial niches. Understanding factors influencing agonist T cells is needed to benefit from their immunoregulatory effects in clinical use.

## Background

Immature T cells, or thymocytes, stem from bone marrow-derived precursors entering the thymus at the junction between the outer cortex and inner medulla of thymic lobules. The subsequent developmental process has been comprehensively studied in mice (1). Following entry, thymocytes are CD4^-^CD8^-^ double negative (DN), and migrate outwards through the cortex towards the subcapsular zone (2). For the αβ lineage, recombination is first initiated at the *Tcrb* locus, resulting in a pre-T cell receptor (TCR) complex consisting of a recombined β chain and an invariant α chain (3, 4). After assessment of the pre-TCR complex at the β-selection checkpoint, the *Tcra* locus of CD4^+^CD8^+^ double positive (DP) thymocytes is recombined (5, 6). Alternatively, thymocytes may diverge into the γδ T cell lineage (7).

DP thymocytes move back through the cortex towards the medulla and go through positive selection (8). This results in death by neglect of thymocytes lacking affinity towards self-peptides presented on Major Histocompatibility Complex (MHC) molecules by cortical thymic epithelial cells (cTECs) (9, 10). A second checkpoint termed negative selection occurs largely in the medulla, when thymocytes have reached the CD4^+^CD8^-^ or CD4^-^CD8^+^ single positive (SP) stage. Tissue-specific antigens produced by medullary thymic epithelial cells (mTECs) may be presented by mTECs themselves, but may also be cross-presented by professional antigen-presenting cell (APC) populations such as dendritic cells (DCs) (11, 12). Peripheral antigens can also be presented by migratory DC subsets circulating to the thymus (13). For thymocytes exhibiting strong responsiveness toward the presented self-antigens, one possible outcome would be induction of apoptosis (14).

However, thymocytes exhibiting reactivity towards self-peptides may alternatively diverge towards agonist selected T cell lineages, including CD8αα T cells and regulatory T cells (T_regs_). The tolerogenic function of agonist selected T cells has led to a substantial interest in their potential for therapeutic use (15, 16). However, uncertainty remains regarding when during thymocyte development divergence into agonist selected lineages occurs, and which factors influence the lineage decision (17–19). While the affinity of the TCR for presented peptide:MHC complexes has been highlighted as a contributing factor, conflicting evidence regarding the TCR affinity of agonist selected lineages could imply additional layers of regulation (20–24).

The thymocyte response upon encounter of specific antigen requires both recognition of peptide:MHC complexes by the TCR (signal 1), and co-stimulatory signalling (signal 2). Co-stimulatory signalling has been attributed crucial roles during T_reg_ development (25, 26). Stromal and APC populations also produce cytokines, which regulate thymocyte migration in a developmentally timed manner (8, 27–29).

To gain insights into the human thymus, we and others have previously studied gene expression in selected thymic cell populations (30–36). Through the Human Cell Atlas initiative, datasets at single-cell resolution for human tissues are rapidly emerging (37). This resource includes single-cell RNA sequencing data of the human thymus across age groups (38). However, elucidation of heterogeneity among thymic APCs and stromal cells is challenging due to their low abundance, and confounded by the profound effects of ageing and thymic involution. Thymic output is at its highest in early infancy, however, loss of thymic mass and structure during ageing results in a corresponding decline in thymocyte production and egression (39, 40). Finally, as the developmental progression of thymocytes is intricately entwined with migration through distinct microenvironmental niches, there is a need to complement insights gained from dissociated samples with methodologies providing spatial information.

Here, we assess human paediatric thymic samples by Cellular Indexing of Transcriptomes and Epitopes by sequencing (CITE-seq) and spatial transcriptomics. In particular, as a result of a carefully developed enrichment strategy, we provide insights into how distinct subsets of thymic APC and stromal cell populations influence agonist selected thymocyte lineages.

## Results

### Single cell profiling of human paediatric thymic samples

**Figure 1:**
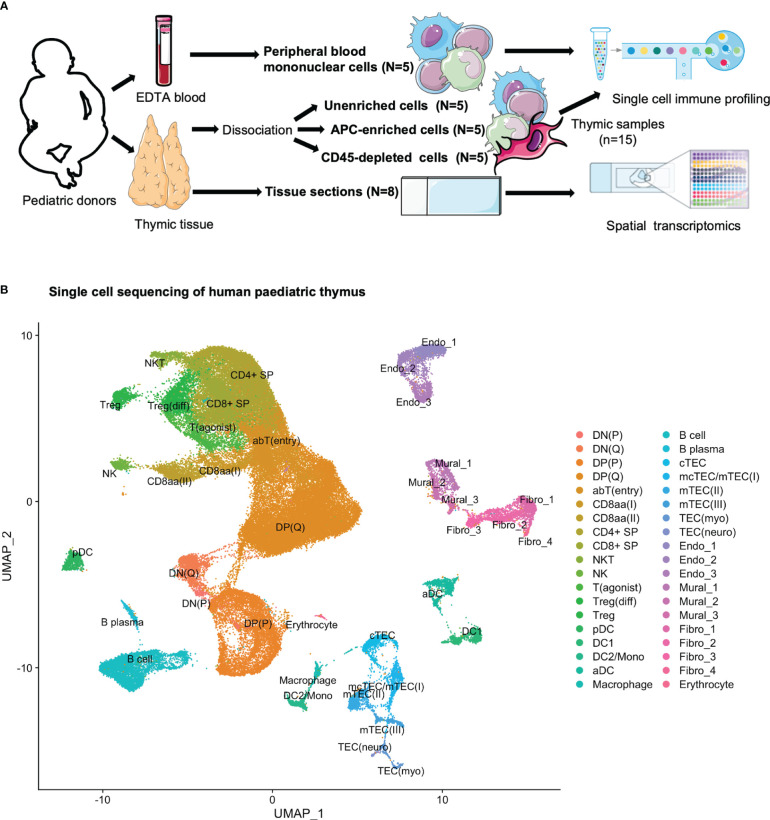
Multimodal profiling of human paediatric thymus. **(A)** Experimental set-up. Tissue was subjected to dissociation (N=5) or snap frozen (N=8) for sectioning. From donors where tissue was to be dissociated, EDTA blood was also collected. CITE-seq was performed for PBMCs (n=5) and thymic cells (n=15) at three separate stages of dissociation: 1. unenriched, 2. APC enriched, and 3. CD45-depleted. Tissue sections were used for spatial transcriptomics. **(B)** UMAP of 83 847 cells colour-coded for the cell types identified in thymus.

Human paediatric thymic tissues (N=5) were dissociated (**Supplementary Table S1**). In order to cover both thymocytes and scarce populations of APCs and non-hematopoietic stromal cells, CITE-seq was performed for three samples per donor: (i) prior to enrichment, (ii) after enrichment for APCs using density gradient centrifugation, and (iii) after enrichment for stromal cells through depletion of CD45-expressing cells (**Figure 1A**; **Supplementary Figure S1**; **Supplementary Table S2**). After filtering, 83 847 cells were retained, and all three thymic samples from all five donors were integrated by use of the integration anchor approach implemented in Seurat (41) for a joint downstream analysis. Through manual annotation, according to expression patterns of established marker genes and genes found to be differentially expressed across populations in previously reported single-cell studies, we identified 38 cell populations and developmental stages, including thymocytes, DCs, B cells, thymic epithelial cells (TECs), fibroblasts, endothelial cells and mural cells (**Figure 1B**; **Supplementary Table S3**).

The distribution of events across each donor was assessed, with no large differences observed (**Supplementary Figure S2A**). In order to assess how the distribution of annotated cell populations varied across the applied enrichment steps, we assessed the contribution of cell types grouped as “early thymocytes”, “late thymocytes”, “agonist thymocytes”, “hematopoietic_APC”, “non-hematopoietic stromal”, and “other” (**Supplementary Figures S2B, C**), indicating an increase in late and agonist thymocytes in addition to APCs after density gradient centrifugation, and an increase in non-hematopoietic stromal cells after CD45-depletion. Correspondingly, while unenriched samples consisted largely of thymocytes exhibiting both RNA and protein level expression of CD3 (**Supplementary Figure S2D**), the APC-enriched and CD45-depeleted samples exhibited an increased proportion of *HLA-DRA*-expressing cells (**Supplementary Figure S2E**). The CD45-depleted samples exhibited an increase in stromal cell populations lacking expression of *PTPRC*/CD45, both at RNA and protein level (**Supplementary Figures S2B, E, F**). However, the protein level data did appear to suffer from high background staining, a previously noted challenge with the CITE-seq approach (42).

### Conventional thymocyte populations followed the established developmental trajectory along pseudotime

**Figure 2:**
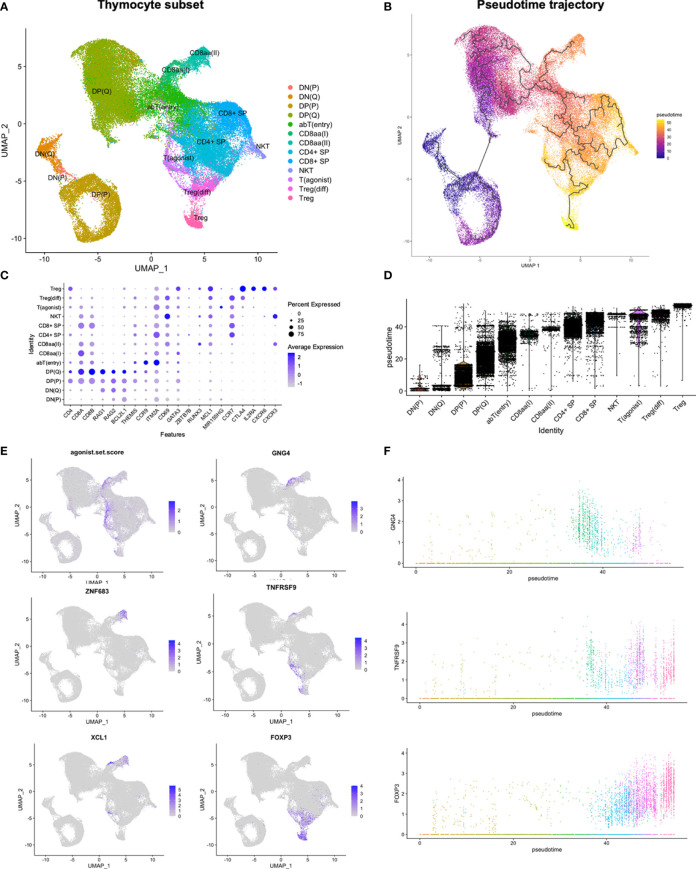
Reanalysis of thymocyte subset. **(A)** UMAP of thymocyte subset. **(B, D)** Pseudotime analysis by Monocle3. **(C, E)** Expression of selected genes in thymocyte subset. The agonist.set.score reflects the mean expression of *NR4A1, NR4A3*, *NFKBID, NFATC1, BCL2L11* and *PDCD1.* **(F)** Expression of selected genes in thymocyte subset along pseudotime. Colouring according to annotations in **(A)**.

To explore the development and lineage divergence of thymocytes in more detail, the thymocyte compartment, consisting of 62 827 cells annotated as DN(P), DN(Q), DP(P), DP(Q), αβT(entry), CD8αα(I), CD8αα(II), T_(agonist)_, T_reg_(diff), T_reg_, or NKT cells, was reanalysed. Due to the substantial influence of cell cycling on the proliferating (P) DN and DP populations, analysis was performed both without and with regression of cell cycle effects, which should mitigate the impact of the cell cycling-associated transcriptional signature upon creation of a low-dimensional representation of the data (**Figure 2A**; **Supplementary Figures S3A, B**). In order to account for the gradual progression of thymocytes through distinct developmental stages, pseudotime analysis was performed by Monocle3, which infers a developmental trajectory based progression through a learned sequence of transcriptional changes (43) (**Figure 2B**; **Supplementary Figure S3C**). Further analysis was performed on the subset without cell cycle regression (**Supplementary Table S4**).

Overall, thymocytes followed the established development from DN, to DP, to SP, defined by both RNA and protein level expression of CD4 and CD8 (**Figure 2C**; **Supplementary Figure S3A**). Quiescent (Q) DP thymocytes expressed *RAG1*/*2* and anti-apoptotic *BCL2L1. THEMIS*, associated with responsiveness to TCR stimulation (44), was upregulated in late DP(Q) and retained through a stage resembling the previously described αβT(entry) cells (*CCR9, ITM2A, TOX2, CD69*) (38) (**Figure 2C**; **Supplementary Figure S4A**). Finally, *ZBTB7B-*expressing CD4^+^ SP cells were predicted to precede *RUNX3*-expressing CD8^+^ SP cells in pseudotime, and both SP populations upregulated anti-apoptotic *MCL-1* (**Figures 2C, D**; **Supplementary Figure S4A**).

Taken together, this suggests a threefold division of human DP thymocytes into stages of proliferation, recombination and selection, in agreement with observations from mice (45, 46). Furthermore, we detected transcriptional changes in genes associated with TCR responsiveness and apoptosis during thymocyte development (**Figure 2C**; **Supplementary Figure S4A**), consistent with a model with variable survival ability upon encounter with specific antigen at distinct developmental time points.

### Thymocytes expressing markers of strong TCR signalling diverged towards agonist selected lineages prior to CD4+ lineage commitment

A subset of cells within αβT(entry) upregulated expression of pro-apoptotic genes and markers of strong TCR signalling (*BCL2L11, NR4A1, NR4A3, NFKBID, NFATC1, PDCD1*) (47–49). In order to assess the summarised expression profile, we calculated an “agonist gene set score” as the mean expression of these six genes. This highlighted a divergence of cells with a high gene set score into two distinct branches. One branch consisted of CD8αα(I) and CD8αα(II) cells according to expression of *GNG4* and *ZNF683*, respectively (**Figures 2A, E, F**). The second branch, termed T_(agonist),_ contained cells at variable developmental time points, spanning cells at earlier stages of development exhibiting the TCR responsive signature, and more mature cells expressing *MIR155HG* and *TNFRSF9* (**Figures 2A–E**; **Supplementary Figures S4B, C**).

We also observed *FOXP3* expression among later-stage T_(agonist)_ cells, with a further gradual increase for the differentiating T_reg_ (T_reg_(diff)) and then T_reg_ populations (**Figures 2E, F**). While the induction of *FOXP3* among the more mature T_(agonist)_ cells would fit with a T_reg_ precursor identity, the inferred pseudotime trajectory failed to predict progression along the T_(agonist)_ branch when analysis was performed without cell cycle regression. Divergence into the T_(agonist)_ branch was predicted to occur at two distinct developmental time points, although both of these observations contrasted with what was predicted upon analysis after regression of cell-cycle effects (**Supplementary Figure S3C**).

Compared to T_reg_(diff), T_reg_ had higher expression of *CXCR6, CXCR3*, and *IL2RA*, and lower expression of *CCR7* (**Figure 2C**). While T_reg_(diff) appeared as more immature than T_reg_, both were predicted to arise subsequently to CD4^+^ SP lineage divergence along pseudotime (**Figures 2D, F**).

Altogether, a transcriptional signature consistent with strong TCR signalling was upregulated prior to lineage commitment of conventional CD4^+^ SP or CD8^+^ SP populations, resulting in deviation from the main thymocyte developmental pathway. While upregulation of genes related to strong TCR signalling was seen at a specific time point, expression of marker genes for distinct agonist selected cell populations and cell states exhibited variable timing of induction.

### Subclusters of activated DCs (aDCs) differed in expression of predicted signalling molecules for interactions with agonist selected thymocytes

The DC compartment, consisting of 3012 DCs, were subsetted out, and reanalysed by re-calculation of highly variable features for creation of a new low-dimensional representation and clustering. These clusters were subsequently used for performing a more fine-grained annotation.

The DC subset included one plasmacytoid (pDC) (*CLEC4C, CD123*) population, two conventional [(DC1 (*CLEC9A, CD141, XCR1*) and DC2 (*CLEC10A, CD1C*)] populations, and an aDC population defined by expression of *LAMP3* and *CCR7* (**Figure 1B**) (50). DC2 was closely associated with monocytes (*CD14, S100A8, S100A9*), resulting in a shared label for these two populations in the initial lower-resolution annotations.

**Figure 3:**
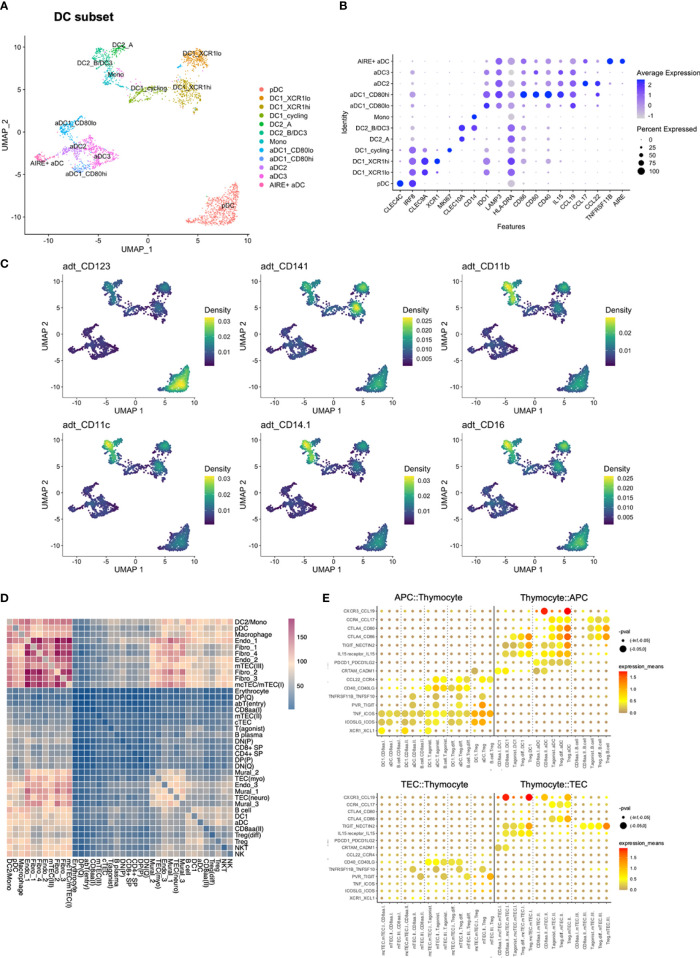
Reanalysis of DC subset and cell-to-cell interactions predicted by cellPhoneDB. **(A)** UMAP of DC subset. **(B)** Expression of selected genes in DC subset. **(C)** Density plot created by Nebulosa showing expression of selected proteins in the DC subset, adt = antibody derived tag. **(D)** Heatmap showing the number of predicted interactions between each combination of annotated cell types/states. **(E)** Expression of selected ligand-receptor pairs in selected pairs of cell types/states. Colour represents the mean of the average expression level of molecule 1 in cell type/state 1, and molecule 2 in cell type/state 2.

After reclustering, DC1 was divided into three clusters, one of which expressed genes related to cell cycling and which was named DC1_cycling (**Figures 3A–C**; **Supplementary Table S5**). Although the DC1 marker *CLEC9A* (51) was expressed in the cycling cluster, expression was reduced relative to remaining DC1 subclusters. The two non-cycling DC1 subclusters differed in *XCR1* and *CADM1* expression, and were termed DC1_XCR1^hi^ and DC1_XCR1^lo^, accordingly (52, 53).

DC1 and pDC clusters expressed *IRF8*, suggested to be involved in the differentiation of DC1, but not DC2 (54). *IRF8* expression was retained in aDC1 and aDC3, which also expressed *IDO1*, reported to mediate tolerogenic effects and to be upregulated upon interaction with T_regs_ (55). However, aDC1 and aDC3 differed by increased *HLA-DRA* expression in aDC1. *CD80* expression was high in aDC3, while aDC1 was spilt into CD80^lo^ and CD80^hi^ subsets. The aDC1_CD80^hi^ subset also exhibited increased expression of *CD40* and *IL15* (**Figures 3A, B**).

The DC2/Mononocyte (Mono) cluster was divided into three subclusters. One pertained to monocytes according to expression of *CD14*, *FCN1*, and inflammatory markers *S100A4* and *S100A11*. The remaining two both expressed DC2 markers *CLEC10A* and *CD1C* (51), and were termed DC2_A and DC2_B/DC3. Among these, DC2_B/DC3 differed from DC2_A by co-expressing the monocyte-associated signature (**Figures 3A–C**).

The aDC2 cluster was defined by high expression of *CCL17* and *CCL22.* Further, aDC2 and DC2_A both expressed *CD1E* and *IL18*, while expression of *TMEM176A* and *TMEM176B* was shared between aDC2 and DC2_B/DC3. Interestingly, while *TNFRSF11B* has previously been reported to be expressed by aDC2s (38), *TNFRSF11B-*expressing aDCs segregated into a distinct cluster. This cluster was termed AIRE^+^ aDC based on expression of *AIRE*, in resemblance to previously reported *AIRE*-expressing, CCR7^+^ DCs (56) (**Figures 3A, B**).

In conclusion, we observed additional heterogeneity among populations of dendritic cells, and observed that the identified subsets exhibited variable expression of several co-stimulatory molecules and chemokines.

### CellPhoneDB predicted interactions between dendritic cells and agonist selected thymocytes

We next assessed cell-to-cell interactions using CellPhoneDB after downsampling the full dataset to 30 000 cells (**Figures 3D, E**). CellPhoneDB is a repository of interacting ligand and receptor pairs, enabling assessment of expression of matched ligand/receptor pairs in order to predict interactions between pairs of cell types.

DC1 was predicted to interact with CD8αα(I) and CD8αα(II) through XCR1-XCL1, as previously reported (38), and the same interaction was predicted for DC1 and T_(agonist)_. The T_(agonist)_, T_reg_(diff), and T_reg_ populations were further predicted to interact with DC1, and to an even larger degree for aDC, through a number of well-established interactions required for T_reg_ development (**Figure 3D**; **Supplementary Figures S5A, B**). Taken together, predicted cell-to-cell interactions by CellphoneDB included several of the signalling molecules that were observed to exhibit differential expression across DC subsets.

### An activated B cell cluster resembled aDC2 in expression of signalling molecules involved in T_reg_ development

**Figure 4:**
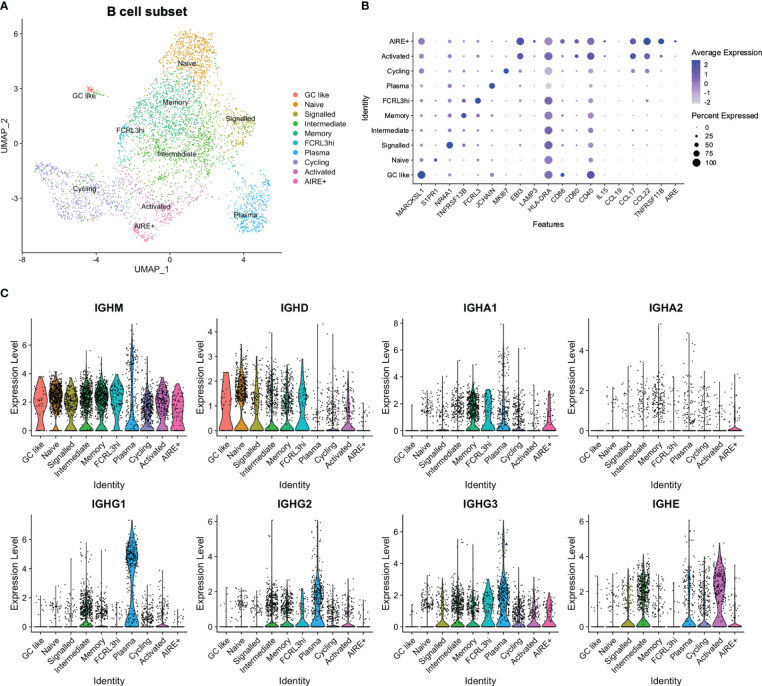
Reanalysis of B cell subset. **(A)** UMAP of B cell subset. **(B)** Expression of selected genes in B cell subset. **(C)** Expression of immunoglobulin genes in B cell subset.

In a similar manner as for the DCs, 4369 B cells (*CD19, MS4A1*) and plasma cells (*CD19, JCHAIN*) were reanalysed in order to obtain a more fine-grained annotation. This revealed clusters largely separated by Ig class, in addition to one cluster defined by cell cycling (**Figure 4**; **Supplementary Table S5**). *IGHM* was widely expressed, while *IGHD*, *IGHA1-2*, and *IGHG1-3* were enriched in specific clusters. One cluster appeared as naïve due to increased frequency of *IGHD* and increased levels of *S1PR1*, where the latter has been implicated in release of immature B cells from the bone marrow and negatively regulated by the activation marker CD69 (57). One cluster was termed “signalled” due to a signature consistent with strong BCR stimulation, including expression of *NR4A1-3* and the CREB*-*modulator *CREM*, although still largely expressing *IGHD.*

Class-switched B cells fell into distinct clusters mainly according to expression of *IGHA1-2*, *IGHE*, or *IGHG1-3*. The *IGHA1-2* enriched cluster appeared to have a memory phenotype according to expression of *CD27* and *TNRFRSF13B* (58), while both *IGHE* and *IGHG1-3* expression was present in a cluster termed “intermediate”. One cluster was identified as plasma cells, with a high abundance of *IGHG1-3* expressing cells, and one *IGHE*-enriched cluster appeared as activated due to expression of *EBI3*, *CD40*, and *CD80*. This activated cluster also expressed *CCL17* and *CCL22*, in resemblance to aDC2. Expression of *CCL17* and *CCL22* was further observed in a cluster defined by expression of *TNFRSF11B*, and which contained a low number of *AIRE*-expressing cells (**Figure 4**).

High expression of *CD40* was also observed in a cluster expressing genes related to germinal centre (GC) B cells (*MARCKSL1*, *NEIL1, MEF2B*) (59–61), which was termed “GC like”. Finally, one cluster expressed *FCRL3*, in resemblance to a previously reported FCRL3^hi^ population (62) (**Figures 4A, B**), although we did not observe the same increase in other reported upregulated genes.

In sum, we identified subsets of B cells, tracking with processes such as BCR activation, Ig class switching, and cell cycling, and identified similarities in expression profiles of signalling molecules between activated B cells and the aDC2 DC subset.

### Subclustering of TECs divided cTECs into two clusters with differential expression of *NEURL2*

**Figure 5:**
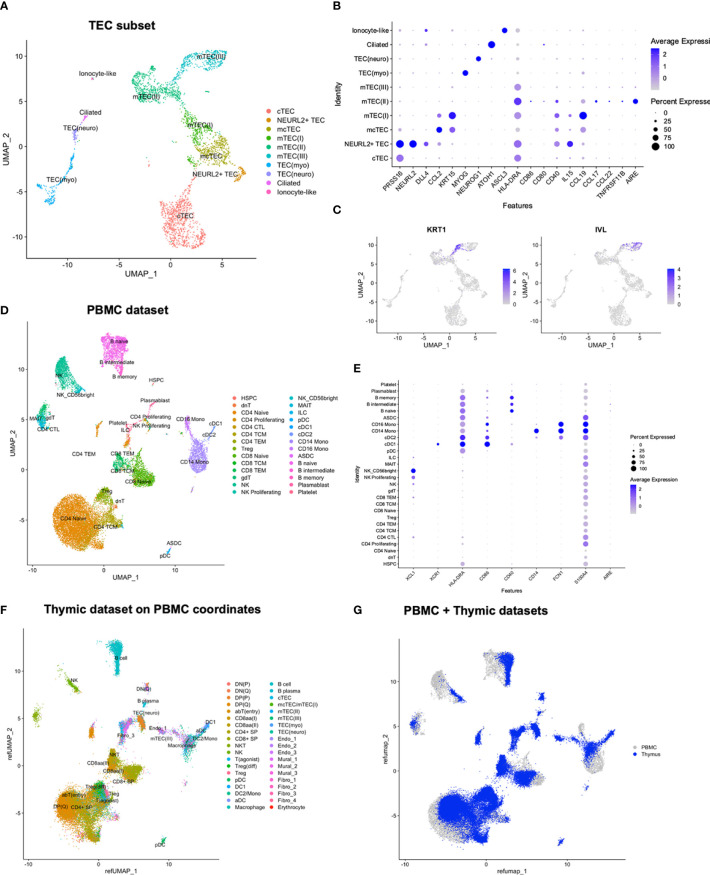
Reanalysis of TEC subset and comparison of thymic and peripheral cell populations. **(A)** UMAP of TEC subset. **(B, C)** Expression of selected genes in TEC subset. **(D)** UMAP of PBMCs with predicted annotations. **(E)** Expression of selected marker genes in PBMCs. **(F)** Thymic dataset mapped onto the UMAP coordinates of the PBMC dataset. **(G)** Merged UMAP of PBMC and thymic datasets after mapping of thymic dataset onto the PBMC UMAP coordinates.

In the full dataset, TECs could be divided into six subtypes: (1) cTEC (*PSMB11*, *PRSS16*, *CCL25*), (2) immature mcTECs/mTEC(I) (*DLK2, CCL2, CCL19, KRT15, CXCL14*), (3) *AIRE-*expressing mTEC(II) (*AIRE, HLA-DRA, NTHL1)*, (4) post-AIRE mTEC(III) (*IVL, ANXA1, ANXA9)*, (5) myeloid TECs (TEC(myo)) (*MYOG, MYOD1, DES)*, and (6) neuroendocrine TECs (TEC(neuro)) (*NEUROG1, NEUROD1, BEX1)* (**Figure 1B**). This subset consisting of 3558 TECs, was also reanalysed (**Figures 5A–C**; **Supplementary Table S5**).

After reanalysis, 10 TEC subtypes were identified, including small subsets expressing ionocyte-like (*FOXI1*, *ASCL3*, *CFTR, CLCNKB*) and ciliated (ATOH1, *GFI1*, *LHX3*, *FOXJ1*) signatures (30). We further highlighted a subset of *PRSS16*-expressing cells diverging from remaining cTECs. This subset, termed NEURL2^+^, was defined by expression of *NEURL2* and *DLL4*, and showed a higher resemblance to mcTEC compared to other cTECs (**Figure 5B**). We noticed a similar divergence of *NEURL2* ^+^ cTECs in two previously published human thymic datasets by Park et al. and Bautista et al. (30, 38) upon reanalysis of TECs from young paediatric samples only (data not shown). Finally, we observed a division between *KRT1^+^* and *IVL* ^+^ cells within mTEC(III), potentially implying unresolved heterogeneity among post-AIRE mTECs (**Figure 5C**).

In order to validate the fine-grained annotations of the TEC subset, we performed label transfer using young paediatric epithelial cells from Park et al. and Bautista et al. (**Supplementary Figures S5C–F**), which largely conformed with manual annotations, apart from a shift in the border between TEC(neuro) and TEC(myo). Overall, this indicated consistent results across datasets when assessing similar age groups, and highlighted the importance of using samples from a narrow age range to fully reveal thymic cell type heterogeneity.

In order to elucidate interactions between agonist selected thymocytes and TECs, we again interrogated the output from CellPhoneDB (**Figures 3D, E**). In resemblance to aDCs, TECs were predicted to mediate interactions with agonist-selected thymocyte populations, with interaction through *CCL19* mediated by the mcTEC/mTEC(I) cluster, in resemblance to aDC1, and interaction through *CCL17* mediated by mTEC(II), in resemblance to aDC2 and activated B cells (**Figure 3E**; **Supplementary Figure S5B**). However, overall, mTEC(II) participated in a relatively low number of predicted interactions, in contrast to what was observed for the mcTEC/mTEC(I) and mTEC(III) clusters (**Figure 3D**).

In addition to supporting previously published TEC subtypes, we identify prospective new subtypes of TECs and pinpoint differences in potential for interactions with agonist selected T cells.

### Medullary and capsular fibroblasts were observed among the non-TEC stromal cells

The non-TEC stromal cells included endothelial cells, mural cells, and fibroblasts (**Figure 1B**, **Supplementary Figure S6**; **Supplementary Table S3**). The initial clustering of the full dataset yielded three endothelial (endo) cell clusters, where Endo_1 and Endo_2 resembled venous endothelial cells in expression of *ACKR1*, and endo_3 resembled arterial endothelial cells (*CXCL12*, *SEMA3G*, and *HEY1*) in addition to expressing *NOTCH4* and *IL32* (63). Lymphatic endothelial cells were scarce, but identified as a small number of cells within Endo_3 expressing *PROX1, FLT4*, and *LYVE1* (63).

Similarly, mural cells were clustered into Mural_1, Mural_2, and Mural_3 (**Figure 1B**; **Supplementary Figure S6**), with Mural_1 having the highest frequency of *MYH11*-expressing cells, indicative of a contractile phenotype. Mural_3 was distinguished in expression of several chemokines, including *CCL21, CCL19* and *CXCL12*.

Among fibroblasts (fibro), four clusters were identified (**Figure 1B**; **Supplementary Figure S6**). Fibro_1 differed from Fibro_2 in increased expression of *COL15A1* and *SFRP2*, while Fibro_2 expressed the highest levels of *IL-33*. Fibro_3 expressed *CXCL9, CXCL10, CD40* and *HLA-DRA*, and Fibro_2 and Fibro_3 expressed *CCL19*, indicating an immune-interacting phenotype, which has been related to medullary fibroblasts (64). Fibro_4 expressed *DPP4, PI16, SEMA3C*, *MFAP5*, and *FBN1*, reported to be expressed by capsular fibroblasts in murine thymus studies, as well as a tissue-universal fibroblast subset in cross-tissue human studies (64, 65).

### Comparison of thymic vs. peripheral cell populations

To compare peripheral and thymic cell populations, we analysed peripheral blood mononuclear cells (PBMCs) from the same five donors from which thymic samples for single cell profiling were taken. After filtering, 19 651 cells were integrated, and annotations were predicted by label transfer from a publicly available CITE-seq reference (66) (**Figures 5D, E**; **Supplementary Figure S7A**). As expected, the predicted annotations included T cells, B cells, NK (Natural killer) cells, DCs, and monocytes.

For a joint visualisation, the thymic dataset was mapped onto the PBMC UMAP coordinates (**Figures 5F, G**). In the resulting plot, CD8αα cells were placed near the peripheral γδ T cells, while T_reg_(diff) was placed near the peripheral T_regs_. Among the T_(agonist)_ cells, some mapped to the peripheral T_reg_ population, while others mapped to the CD4 central memory T cells, further pointing to a differentiation decision fork within the T_(agonist)_ population.

Peripheral T cells and B cells were largely naïve. Unlike thymic samples, *NR4A1-3* expression was not seen among PBMCs. Further, lack of *CCL17* and *CCL22* in PBMCs highlights the relevance of the thymic environment in inducing expression of these signalling molecules. *CD40* exhibited wide expression among peripheral B cells, but was only seen at lower levels among peripheral DCs, consistent with lack of other aDC markers. Peripheral cDC1s matched their thymic counterparts in expression of *XCR1*, and the peripheral cDC2 contained inflammatory, monocyte-like cells as observed for thymic DC2 (**Figure 5E**).

Naïve peripheral T cells widely expressed *ITM2A*, related to thymocyte selection (45). By contrast, expression of *TOX2*, similarly related to αβT(entry) thymocytes, was highly restricted among peripheral T cells, and genes related to strong TCR signalling and agonist selection, including *NR4A1* and *PDCD1*, were not expressed. While *CXCR3* and *CXCR6* have been suggested to be expressed by T_regs_ recirculating to the thymus from the periphery (36), and found to be expressed in mature thymic T_regs_ in our dataset, we did not observe expression of these genes among the peripheral T_regs_. The *XCR1* ligand *XCL1* was mainly expressed by NK cells among the PBMCs, particularly in predicted CD56^bright^ NK cells (**Figure 5E**).

### Spatial transcriptomics mapped *XCL1*-expressing agonist selected populations and *XCR1*-expressing DC1 to the cortico-medullary junction

In order to elucidate the spatial organisation of thymic cell populations, we performed spatially resolved RNA sequencing of eight fresh-frozen paediatric human thymic tissue sections (**Supplementary Figure S7B**) using the Visium spatial transcriptomic solution.

**Figure 6:**
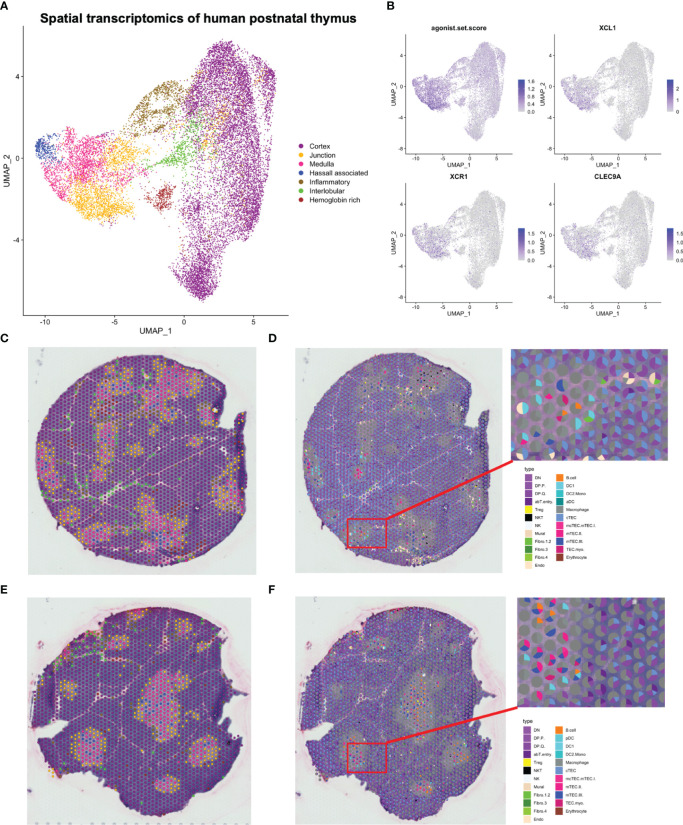
Spatial transcriptomics of human paediatric thymus. **(A)** UMAP of spatial transcriptomics dataset. **(B)** Expression of selected marker genes in spatial transcriptomics dataset. The agonist.set.score reflects the mean expression of *NR4A1, NR4A3*, *NFKBID, NFATC1, BCL2L11* and *PDCD1.* **(C, E)** Representative tissue sections coloured by annotations in **(A)**. **(D, F)** Spatial deconvolution of representative tissue sections by SPOTlight.

Data from all sections, consisting of 18 329 spots, were integrated, and manual annotation of clusters was performed based on both transcriptional profiles and on the spatial localisation of participating spots (**Figures 6A–C, E**; **Supplementary Figures S7C, S8, S9A**; **Supplementary Table S6**). With this approach, clusters pertaining to the cortex, medulla, cortico-medullary junction and inter-lobular zones could be identified. One cluster was termed “Hemoglobin rich” due to high expression of hemoglobin genes, and three clusters that appeared to consist of spots both at the cortico-medullary junction and the inter-lobular region were given the collective term “inflammatory” according to high expression of an inflammatory gene signature. Inflammatory genes were also expressed by a cluster residing within the thymic medulla, which in addition expressed genes related to mTEC(III). This cluster was termed “Hassall associated”, reflecting on the keratinized structures formed by post-AIRE mTECs (67).

**Figure 7:**
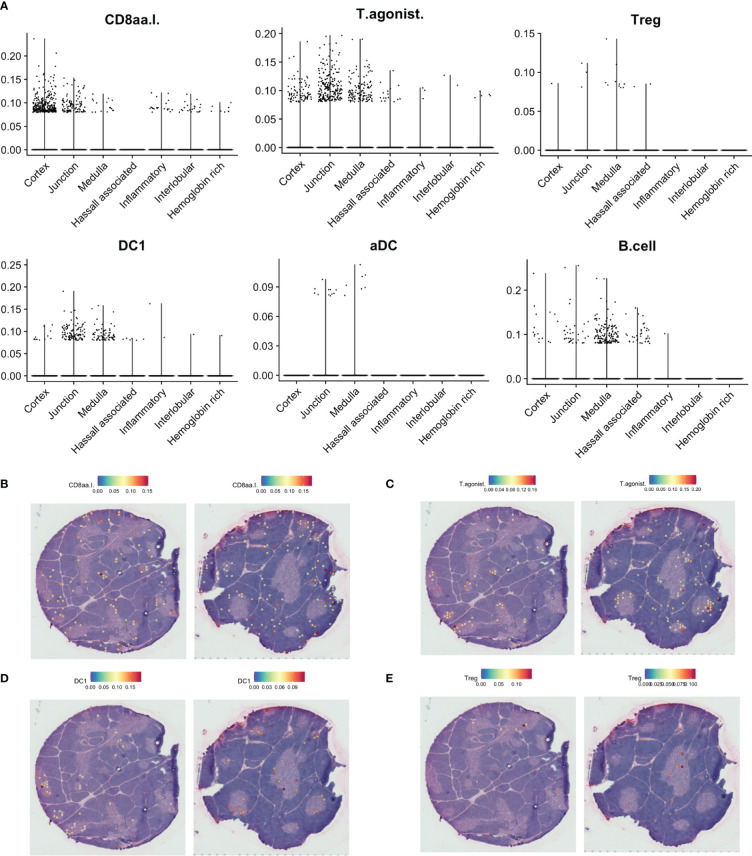
Predicted localisation of selected agonist selected and antigen-presenting cell populations by SPOTlight. **(A)**. Predicted proportion of selected populations among tissue spots. **(B–E)**. Localisation of spots predicted to include selected cell populations on representative tissue sections.

**Figure 8:**
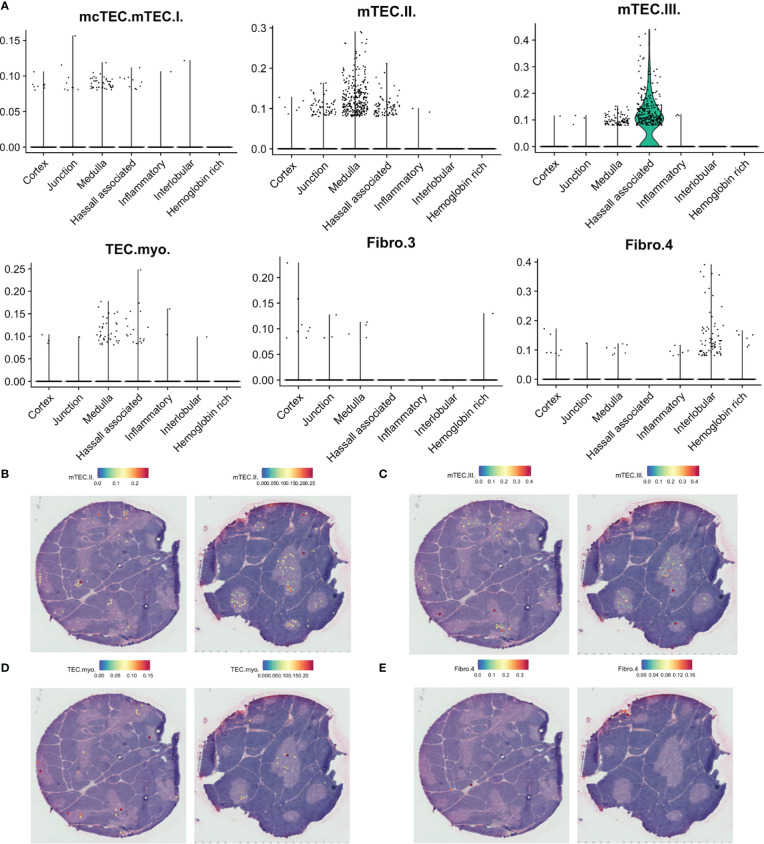
Predicted localisation of selected stromal cell populations by SPOTlight. **(A)**. Predicted proportion of selected populations among tissue spots. **(B–E)**. Localisation of spots predicted to include selected cell populations on representative tissue sections.

To deconvolute spots into their cell-type composition, we used SPOTlight with our single cell RNA sequencing dataset as reference (**Figures 6D, F**). Merging of highly similar clusters was necessary to facilitate building of cell-type specific topic profiles, resulting in 31 uniquely annotated clusters (**Supplementary Figure S9B**). We observed that the cortical spots largely were composed of DP thymocytes and cTECs, while the medulla exhibited a larger cellular heterogeneity and included mTECs, DCs, and B cells (**Figures 6D, F**, **7**, **8**; **Supplementary Figure S10**).

While DC1 was predicted to be present in the medulla, this population was also predicted to be largely present at the cortico-medullary junction (**Figures 7A, D**), fitting with a similar pattern for expression of *CLEC9A* and *XCR1*, and coinciding with expression of *XCL1* and the previously defined agonist gene set score (**Figure 6B**). In contrast, *FOXP3* expression and predicted T_reg_ contribution had a tendency towards medullary spots (**Figures 7A, E**).

Finally, we observe differences in the spatial location of fibroblast subsets. While the suggested tissue-remodeling Fibro_4 population was mainly located to interlobular connective tissue, the suggested immune-interacting Fibro_3 population was found deeper within the thymic lobules (**Figures 8A, E**).

### A hypothetical model of human thymic agonist selection

**Figure 9:**
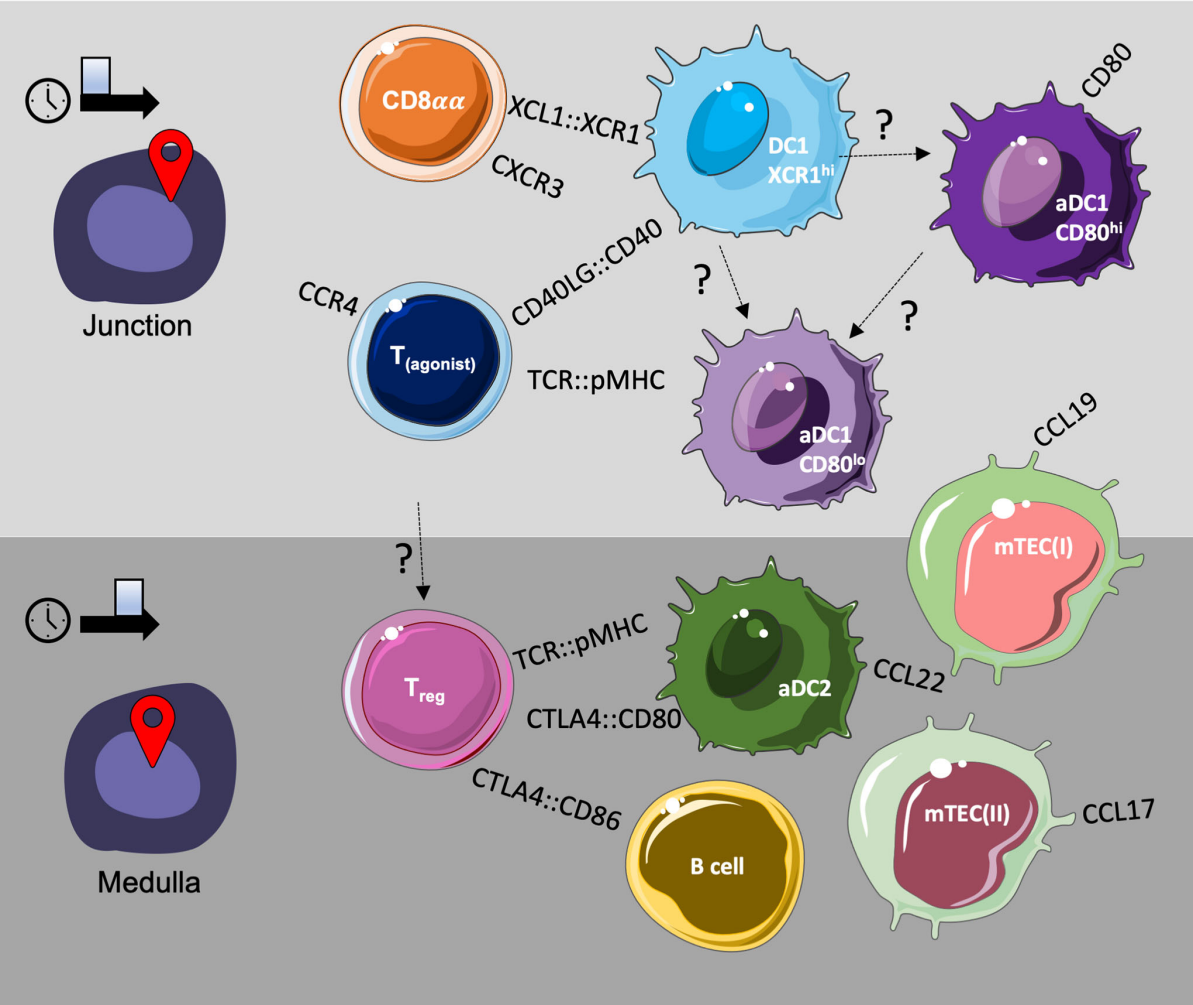
Putative model of the cellular milieu for T agonist selection in the thymus.

Overall, our findings support recruitment of DC1 cells to the cortico-medullary junction by cells undergoing agonist lineage divergence at an earlier developmental time point, while T_reg_ divergence at later developmental stages and T_reg_ maintenance may be supported by APC and stromal cell populations present within the medullary compartment, expressing a distinct profile of signalling molecules. A schematic summary, representing a hypothetical, possible model of the cellular milieu of thymic agonist selection based on the observations from both our single-cell RNA sequencing and spatial transcriptomics datasets, is provided below (**Figure 9**).

## Discussion

The single cell field has evolved towards the inclusion of an increasing number of data modalities. While others have provided single cell RNA sequencing datasets of the human thymus spanning across different age groups (38), or focused on specific cellular compartments (30, 31, 34, 36, 68, 69), we present a comprehensive profiling of samples at a narrow paediatric age range. To this end, we performed spatial transcriptomics of fresh-frozen thymic tissue sections, in addition to CITE-seq of thymic cells sampled at three distinct steps during a gradual enrichment protocol. As a result, we were able to study the developmental trajectory and spatial localisation of thymocytes in concert with the APCs and stromal cells that impose crucial checkpoints and regulatory signalling molecules at specific developmental time points. Our approach not only yielded the expected enrichment for APCs and stromal cells, but also allowed a better overview of thymocytes outside of the otherwise dominating DP(Q) stage. This facilitated exploration of the contributions by non-thymocyte populations to the development and maintenance of agonist selected thymocyte lineages.

The αβT(entry) stage, consisting of cells showing a transcriptional signature associated with T cell activation, appeared to represent a crossroad between the conventional trajectory of CD4^+^/CD8^+^ SP lineages, and divergence towards agonist selected lineages. Two branches leading from the αβT(entry) stage to either a CD8αα or a T_(agonist)_ fate exhibited striking similarities, both showing a gradual shift from a signature indicating strong TCR signalling, to a signature of expected marker genes for the respective lineages. A similar gene signature of strong TCR signalling has previously been attributed to thymocyte agonist selection by Chopp et al. (68), who performed single-cell RNA sequencing on human thymocytes after cell sorting in order to cover pre-selection thymocytes, CD69-expressing thymocytes, and CD4^+^ and CD8^+^ SP thymocytes. In agreement with our findings, divergence of thymocytes undergoing agonist selection was attributed to a developmental time point prior to CD4^+^ or CD8^+^ lineage commitment. However, as in our study, this conclusion was based on inference of a pseudotime trajectory by Monocle3, not by functional experiments. In contrast to our findings, only one agonist-selected branch was identified, which could be due to the employed cell sorting strategy.

The later-stage cells of the T_(agonist)_ branch in our data co-expressed genes related to T_regs_, which would be in agreement with this branch representing a potential T_reg_ developmental pathway. Viewing the T_(agonist)_ population as a series of cell states would agree with their predicted wide spread along pseudotime. Like T_(agonist)_, the T_reg_(diff) cluster exhibited upregulation of T_reg_ marker genes, including *FOXP3*, and represented a possible T_reg_ progenitor population. However, unlike the T_(agonist)_ cells, T_reg_(diff) were predicted at a narrow window along pseudotime, subsequent to CD4^+^ lineage commitment.

In contrast to the division between one early and one mature T_reg_ population reported by Chopp et al. (68), the single cell RNA-sequencing datasets by Park et al. (38) and Morgana et al. (36) report the presence of three distinct T_reg_ and putative T_reg_ precursor populations in the human thymus. Morgana et al. performed cell sorting prior to sequencing in order to cover specific thymocyte subset, while the Park et al. dataset includes samples from a variety of unenriched or enriched samples, with enrichment largely focusing on increasing the coverage of CD45^-^EpCAM^+^ epithelial cells.

Based on their observations, Morgana et al. propose a model of continuous development from a population resembling T_(agonist)_, to a population resembling T_reg_(diff). In contrast, Park et al. propose a model of two distinct developmental pathways, concordant with findings from mice (70). Further, Morgana et al. suggest that their most mature cluster represent recirculating T_regs_ entering the thymus from the periphery. However, the identification of human recirculating T_regs_ remains challenging due to lack of established markers. While we observe that our most mature thymic T_reg_ population resembles the proposed recirculating T_regs_, including high expression of *CXCL3* and *CXCL6*, a similar signature was not seen for our peripheral T_regs_.

While we cannot exclude the possibility that T_reg_(diff) represents an intermediate T_reg_ precursor state originating from T_(agonist)_, our inferred pseudotime trajectory indicates T_reg_(diff) to originate from the CD4^+^ SP population. Low expression of *IL2RA* was observed among both putative T_reg_ precursor populations in our dataset, which, together with the observation of surface CD25 protein in only one out of the two putative T_reg_ precursors described in mice, underlines the concern raised by Morgana et al. regarding their use of CD25 as a marker for cell sorting prior to single cell sequencing.

A noteworthy difference between the two putative T_reg_ precursors in our dataset was the presence of CD4^+^CD8^+^ DP cells among T_(agonist)_ but not T_reg_(diff). Although the majority of T_regs_ are derived from mature CD4^+^ SP thymocytes in murine models, the ability of human DP cells to upregulate *FOXP3* upon co-culture with allogeneic primary TECs has been demonstrated (71). Thus, it could be speculated that the T_(agonist)_ branch, in contrast to T_reg_(diff), demarcate a pathway towards a T_reg_ fate inducible by high-affinity TCR interactions prior to commitment into the CD4^+^ SP or CD8^+^ SP lineages, resembling development towards CD8αα cells.

Overall, these findings would be in agreement with the notion that CD8αα lineage commitment occurs at a stringently defined developmental time-point and requires high-affinity TCR interactions with presented peptide:MHC complexes (18, 19, 72). In contrast, T_regs_ may be derived from thymocytes at variable developmental time-points and exhibit a wide range of TCR affinities (21, 71). However, further studies are needed to elucidate whether T_(agonist)_ represents a continuous pathway distinct from conventional T cell development after an initial branching, or whether thymocytes may diverge from the conventional T cell development towards the T_(agonist)_ branch at distinct developmental time points. In our hands, inference of a pseudotime trajectory graph for divergence and progression of T_(agonist)_ cells provided different predictions upon analysis with or without regression of cell cycle effects. There is also a need to assess how divergence of T_(agonist)_ cells relates to TCR affinity and to the variation in TCR responsiveness observed during the course of thymocyte maturation (44, 73).

It is further worth noting that, in our data, cells among the T_(agonist)_ population, but not T_reg_(diff), expressed *XCL1*, which has previously been reported in CD8αα cells and terminally differentiated mTECs. *XCL1* has been suggested to act in the recruitment of the cross-presenting *CLEC9A* ^+^ DC1 population (38, 74). While APCs have been reported to favor a medullary localisation in the human thymus (67), our spatial analyses indicated that the transcriptional signature associated with strong TCR stimulation and subsequent agonist selection was located largely to the cortico-medullary junction. An active recruitment could ensure the availability of DC1 cells at the same location, and indeed, DC1 were predicted to reside both at the cortico-medullary junction and within the medullary compartment in our spatial transcriptomics dataset. However, the lack of single-cell resolution of the Visium technology necessitated computational deconvolution in order to make inferences regarding the cellular composition of capture spots, and uncertainty regarding the quality of the predicted annotations exists, in particular for several of the highly similar thymocyte populations. Still, the predicted localisation of DC1 appeared to fit with expression of *CLEC9A*, and would also be in agreement with previous reports containing spatial transcriptomics data from the human fetal thymus, highlighting robustness across studies and applied deconvolution methods (75). DC1 could then mature towards the aDC1/aDC3 states, induced in part by interacting with thymocytes through the CD40-CD40LG axis. Binding of CD40 to CD40LG has been reported to mediate activation and “licensing” of APC populations, enhancing their capability for driving T_reg_ generation (26, 76).

The presence of an aDC1_CD80^lo^ population, exhibiting reduced expression of *CD80*, would be of interest in light of studies highlighting the role of CD80^neg^/^low^ hematopoietic APCs in the development of thymic IEL precursors in mice (77). By contrast, aDC1_CD80^hi^ expressed comparably higher levels of *CD80* and also exhibited the highest levels of *IL-15* expression among the DCs, reported to be involved in a second, TCR-independent step of T_reg_ generation in mice, occurring after initial TCR stimulation (78). Such secondary signalling can alternatively be provided by IL-2, and IL-2 availability has been suggested to represent a potential negative feedback mechanism regulating the size of the T_reg_ niche. A differential dependency on IL-15 and IL-2 between the two putative T_reg_ precursors in mice have been suggested (79), presenting an intriguing possibility that the IL-2 feedback loop also affects the progenitor populations differently.

Expression of *CD80* and *CD40* was also seen among thymic B cells, co-occurring with expression of *CCL17* and *CCL22* in a cluster enriched in IgE class-switched B cells. These two chemokines have been reported to be upregulated upon stimulation by Hassall-derived TSPL, and to drive the upregulation of *FOXP3* in CD4^+^CD25^-^ precursor cells (26, 80). As such, this could imply a function in divergence of later-stage, CD4^+^ lineage-committed thymocytes toward a T_reg_ fate within the medulla. In agreement, the highest expression levels of *CCL17* and *CCL22* in our spatial dataset pertained to medullary spots. However, it should be noted that lower-level expression was also evident among spots locating to the cortico-medullary junction, and that expression of the *CCR4* receptor mainly related to the earlier-diverging, strongly TCR-signalled agonist selected populations.

A *CCL17* ^+^ *CCL22* ^+^ subset has previously been described in a study employing single cell RNA sequencing on CD21^-^CD35^-^CD19^+^ cells from human postnatal thymus (31). The same study further reported a proliferating cluster, also in agreement with our data, and argued that these proliferating B cells differentiated into plasma cells. Intriguingly, this suggested developmental trajectory was further supported by *in vitro* differentiation of purified cells corresponding to the proliferating B cell cluster. In contrast to our findings, however, this study reported T-cell engagement mainly in a separate *CD80*/*CD86*-expressing cluster that was not observed in our study.

Expression of *CCL17* and *CCL22* was also found to be higher in aDC2 in our data compared to the *CD40-* and *CD80-*expressing aDC1. This could imply potential division of labor among aDCs, fitting with lack of *XCR1* for junctional recruitment on DC2, the suggested aDC2 precursor (38). However, this suggested lineage relationship was derived from summarised marker gene expression profiles rather than functional experiments, and is further confounded by our observation of heterogeneity among DC2, with one subset expressing a monocyte-like signature, in agreement with findings from the periphery in human studies (51, 81). Whether the non-inflammatory and inflammatory subsets are derived from a common DC lineage has been a matter of debate, although evidence in favor of a separate developmental trajectory has led to the suggestion that the inflammatory subset should be given the independent designation “DC3” (82). In our hands, some similarity to aDC2 was observed for both non-inflammatory DC2_A and inflammatory DC2_B/DC3, but further studies are needed to clarify their relationship to aDC2.

Both the DC and B cell subsets contained *TNFRSF11B^+^AIRE^+^* clusters, resembling the aDC2 and the activated B cell cluster, respectively. However, although the presence of *AIRE-*expressing human thymic B cells would agree with previous reports (83), the number of *AIRE^+^* B cells was low, and these cells should be interpreted with caution as they may represent doublets according to high doublet scores.

Intriguingly, among the mTECs, expression of *CCL22* and *CCL17* was associated with the AIRE^+^ mTEC(II) population. The AIRE^-^ mcTEC/mTEC(I) and mTEC(III) populations, in contrast, appeared to provide a repertoire of signalling molecules more similar to aDC1, including expression of *CCL19*. We further note a substantial overall resemblance between the two *AIRE^-^* mTEC clusters, mcTEC/mTEC(I) and mTEC(III). However, TEC(III) was defined by expression of an inflammatory signature, which would be in agreement with previous evidence in favor of the Hassall-forming mTEC(III)s also aiding T_reg_ generation by maintaining an inflammatory milieu (67, 80, 84, 85). Expression of *KRT1* and *IVL* localised to distinct subsets within the mTEC(III) cluster, which could imply additional heterogeneity, possibly denoting the transition between mTEC(II) and mTEC(III).

Several genes common to mcTEC/mTEC(I) and mTEC(III) were also expressed by *NEURL2^+^* cTECs. However, the relevance of *NEURL2^+^* cTECs remains to be elucidated. *NEURL2* encodes an E3 ubiquitin ligase, and among its substrates is β-catenin, which mediates signalling through the canonical Wnt signalling pathway and has been found to be crucial for commitment of embryonic endodermal epithelial cells to a thymic cell fate (86–89). The *NEURL*2^+^ cluster also expressed *DLL4*, implicated in signalling to Notch1 during lineage commitment of T cell progenitors in mouse (90), and reported to progressively decrease with age. Widespread expression of *NEURL2* in previously published embryonic cTEC further suggests an age-dependent effect, which is of interest as changes in regulation of TEC differentiation and function during ageing and onset of thymic involution has been observed (91).

Immunoregulatory signalling was also observed for non-TEC stromal populations, in particular Fibro_3. An immune-regulatory function has previously been attributed to a medullary thymic fibroblast subset in mice, identified by a gradual digestion of thymic tissue developed in order to achieve a physical separation of capsular and medullary fibroblasts. The medullary fibroblasts were suggested to provide cell type-specific antigens for presentation to developing thymocytes (64, 92). By contrast, capsular thymic fibroblasts, which resemble the Fibro_4 population in our data, has been reported to have a tissue-remodeling function (64, 93). Fibro_4 further resembled the mouse tissue-universal *Pi16* ^+^ fibroblast population, suggested to be capable of developing into tissue-specific, functionally differentiated subsets. In a cross-tissue mouse study of fibroblast subsets, differentiation was suggested to occur by progression through another tissue-universal population denoted by expression of *Col15a1* (65), a feature which is shared by Fibro_1 in our dataset. However, differentiated fibroblast subsets arising subsequently to the *Col15a1* population appeared to exhibit substantial cross-tissue variation, and as thymic tissue was not represented, this would confound comparisons to populations identified in our study.

In summary, the present work expands current knowledge about thymic agonist selection and signalling provided by subpopulations of thymic APCs and stromal cells. We define a branch point towards a CD8αα or T_(agonist)_ fate occurring at the cortico-medullary junction, coinciding with a highly TCR responsive transcriptional signature and signalling for recruitment and activation of DC1. Subsets of aDCs, possibly derived from DC1 and DC2, defined by distinct profiles of signalling molecules, might point toward roles in separate thymic compartments. Intriguingly, similarities between aDC2, activated B cell, and mTEC(II) were observed. However, their effects on thymocytes diverging towards agonist selected T cell lineages at distinct developmental time points remains to be elucidated. The gained knowledge regarding development and maintenance of agonist selected T cell lineages would be of immense value in the effort to benefit from the immunoregulatory potential of such populations in clinical applications.

## Methods

### Human thymic tissue and blood samples

Thymic tissue was obtained from young paediatric patients (8 male, 5 female, age span 7 days-13.5 months) undergoing corrective cardiac surgery at the department of cardiothoracic surgery, Oslo University Hospital. In cases where tissue was to be used for single cell immune profiling, 4 ml EDTA blood was sampled from the same patient. The project was approved by the Regional Ethics Committee of South East Norway (REC 31516), and conducted in compliance with the Declaration of Helsinki. Written informed consent was obtained from the parents of the patients. Patients exhibited congenital heart disorders but no other known medical conditions potentially affecting autoimmune risk.

### Isolation of peripheral blood mononuclear cells

Peripheral blood mononuclear cells were isolated by LymphoPrep™ (Abbott) in SepMate tubes (Stemcell Technologies) according to protocol by Stemcell Techonologies. Isolated cells were subjected to red blood cell removal by the EasySep™ RBC Depletion Reagent (Stemcell Technologies).

### Dissociation of thymic samples

Tissue was placed in RPMI-1640 (Sigma-Aldrich) with 10% fetal bovine serum (FBS) on ice immediately after surgical removal, and processing was initiated shortly (30-60 min) after. Connective tissue, blood clots and necrotic tissue were removed before cutting the tissue into 2-4 mm pieces. Tissue pieces were subjected to three rounds of pipetting with a widened pipette tip in RPMI-1640, each time removing the supernatant after allowing tissue pieces to settle. Supernatants from the two last rounds were pooled, filtered through a 70 μm filter, and set aside on ice.

Tissue pieces were subjected to five rounds of enzymatic dissociation using Liberase™ TM. For each round, tissue was distributed across 2-4 gentleMACS™ C tubes (Miltenyi Biotec) in 10 ml enzyme cocktail (RPMI-1640, Liberase™ TM 0.17 U/ml (Sigma-Aldrich), DNaseI 0.1% w/V (Sigma-Aldrich), and placed on a gentleMACS™ Octo Dissociator (Miltenyi Biotec) at 37 °C. Incubation time was 15 min for round 1-4, and 5-30 min for round 5, depending on the amount of undissolved tissue. Further details regarding the gentleMACS™ program are available upon request. C-tubes were centrifuged at 100 x g at room temperature for 30 s. The pellet of undissolved tissue was subjected to a new round of dissociation, while the supernatant (containing released cells) was collected and mixed 1:1 with resuspension buffer (PBS, FBS 5%, EDTA 5 mM, DNaseI 0.1% w/V), before filtration through stacked 70 μm and 30 μm filters and centrifugation 350 x g 4 °C 10 min. The pellet was resuspended in 5-10 ml resuspension buffer and set aside on ice. Released cells from all five rounds were pooled and filtered through a 70 μm filter.

An unenriched sample was set aside, containing 6 x 10^6^ cells kept from the pipetting steps before enzymatic digestion, and 6 x 10^6^ cells kept from the released cells during enzymatic digestion in Liberase™ TM.

Debris remaining after Liberase™ TM dissociation was resuspended in 2.5 ml enzyme cocktail supplemented with 0.25% Trypsin-EDTA (Thermo Fisher Scientific), to a final concentration of 0.05% Trypsin-EDTA (94) and placed in a C tube on a gentleMACS™ Octo Dissociator, and incubated at 37 °C for 45 min, details regarding the gentleMACS™ program available upon request. Released cells were filtered through stacked 70 μm and 30 μm filters, centrifuged 340 x g for 10 min, and resuspended in 5 ml resuspension buffer.

The unenriched sample and cells released during trypsin dissociation were subjected to red blood cell removal as described for PBMC samples. Unenriched sample was subjected to dead cell removal by use of Dead Cell Removal Kit (Miltenyi Biotec).

### Enrichment by density gradient centrifugation and CD45 depletion

Cells released during Liberase™ TM dissociation were subjected to OptiPrep™ (Abott) density gradient centrifugation. Cells were centrifuged 340 x g at 4 °C for 10 min, resuspended in resuspension buffer, and centrifuged as before. 1.070 g/ml and 1.061 g/ml gradient solutions were prepared by mixing OptiPrep™ solution with appropriate volume of dilution buffer (MilliQ H_2_O, NaCl 0.8%, EDTA 5 mM, Tricine-NaOH 10 mM, pH 7.4), and supplementing with 0.1% w/V DNase1. Cell pellet was resuspended in 5 ml 1.070 g/ml gradient per 1 x 10^9^ cells, and 5 ml suspension was transferred to the required number of 15 ml conical tubes. Next, 5 ml of the 1.061 g/ml gradient per tube was added on top of the 1.070 g/ml gradient, followed by a layer of 2.5 ml FBS.

Tubes were centrifuged at 1700 x g 4 °C for 30 min, with brake and acceleration at lowest possible setting. A band of cells residing at the top of the 1.061 g/ml layer was collected, washed in PBS, centrifugation 340 x g 4 °C for 10-15 min, and resuspended in PBS.

Cells collected from OptiPrep™ gradient enrichment were pooled with cells from trypsin dissociation, and subjected to dead cell removal by use of Dead Cell Removal Kit as before. An APC-enriched sample of 200 000 cells was set aside. Remaining cells were subjected to CD45 depletion by use of the EasySep™ Human CD45 depletion kit II (Stemcell Technologies), and 200 000 cells were set aside as the CD45-depleted sample.

### CITE-seq

200 000 cells from each sample were resuspended in 50 μl staining buffer (PBS, FBS 2%) before addition of 5 μl Human TruStain FcX™ Blocking reagent (Biolegend) and incubation for 10 min at 4°C. 50 μl Antibody pools as indicated in **Supplementary Table S2** (further details available upon request) were centrifuged at 14 000 x g 4 °C for 10 min and added to the samples, followed by incubation for 30 min at 4°C. Samples were washed three times in 1.5 ml PBS w/1% BSA, centrifugation 400 x g for 5 min at 4 °C. Cells were resuspended in 200 μl resuspension buffer (PBS, BSA 0.04%) to a concentration of 1000 cells/μl. Samples were subsequently processed according to the Chromium Single Cell 5’ V(D)J Reagent Kit User guide with Feature Barcode Technology for Cell Surface Protein, v1 Chemistry (10x Genomics protocol CG000186, RevD), aiming to recover 10 000 targeted cells. The cDNA amplification was run with 13 cycles for PBMC and unenriched samples, and 15 cycles for the APC-enriched and CD45-depleted samples. For the feature barcoding sample indexing PCR, 6 cycles were run for the unenriched and APC-enriched samples, and 9 cycles were run for the PBMC and CD45-depleted samples.

Sequencing was performed on Illumina NovaSeq S2 flow cell, 100 cycles. According to recommendations by 10x Genomics, a sequencing depth of at least 20 000 read pairs per cell was obtained for the gene expression libraries, and at least 5000 read pairs per cell for the antibody capture libraries.

### Spatial transcriptomics

Thymic tissue was collected as previously described. Tissue was cut into pieces of about 1 cm x 5 cm x 2 cm and snap frozen using liquid nitrogen, embedded in Optimal Cutting Temperature (OCT) reagent, before storing at -80 °C until further processing. Ten 10 μm sections of tissue were collected from each of the frozen tissue samples, and RNA was extracted by the Norgen Bio kit (RNA/DNA/Protein Purification Plus Kit, #47700) to control for the RNA integrity. Sections for hematoxylin and eosin (H&E) staining were performed simultaneously. Only tissue with RIN>7 and good quality H&E staining was used for the Visium Spatial Transcriptomics solution. After optimising the cryosectioning and permeabilisation time, 6 mm pieces of 10 μm were punched out of tissue pieces stemming from eight separate paediatric donors and placed in capture areas on 10x Genomics Visium Spatial gene expression slides. Slides were stored at -80 °C until library preparation (max 4 weeks). Slides were fixed in methanol, followed by H&E staining (10x Genomics protocol CG000160, rev B) and imaged by a LSM800 confocal laser scanning microscope. Next, slides were permeabilised for 12 min, and library preparation was performed according to the Visium Spatial Gene Expression Reagent User Guide (10xGenomics protocol CG000239, rev.D). All incubations and PCRs were performed on a Veriti thermal cycler (Thermo Fisher Scientific). Sequencing was performed on a Illumina NovaSeq SP flow cell, 100 cycles, to a sequencing depth of 32 000 - 83 000 read pairs per tissue covered spot.

### Data analysis

For CITE-seq experiments, the raw sequencing data were processed by the 10x Genomics CellRanger v.3.1.1 pipeline. For the spatial transcriptomics data, manual alignment of fiducials was performed using Loupe browser v.5.1.0 (95), before running the 10x Genomics SpaceRanger v.1.2.2 pipeline. The sequences were aligned to the GRh38-2020-A reference genome. Further analysis was performed using Seurat v.4.0.3/4.0.4 (41, 66, 96, 97) under R v.4.0.3/4.1.0-4.1.3 (98). Quality control statistics (**Supplementary Table S7**) were calculated according to the filtered CellRanger or SpaceRanger output count matrices, where empty droplets are removed.

#### Preprocessing, integration, and annotation of the full thymic RNA sequencing dataset

For thymic samples (unenriched, APC-enriched, and CD45 depleted samples), ambient RNA correction was performed using SoupX v.1.5.2 (99) and doublet scores were calculated by scDblFinder v.1.6.0 (100). Features detected in < 3 cells, and cells with mitochondrial genome content >10% and number of detected features < 500 or > 5500 were removed. For gene expression data, log normalisation was performed, while antibody capture data were normalised by Seurat’s centered log ratio (CLR) approach. Variable features were defined per sample as the top 2000 variable features from the RNA assay. Integration of all thymic samples from all five donors was performed by the reciprocal Principal Component Analysis (RPCA)-based integration anchor workflow implemented in Seurat. The integrated data were scaled, and number of detected features, number of counts, and percentage of mitochondrial content was regressed out. Dimensionality reduction was performed first by PCA, and subsequently on the first 50 principal components by Uniform Manifold Approximation and Projection (UMAP) (101). Clustering was done using the Louvain algorithm with a range of resolutions from 2.2 to 4.0. Manual cell type annotations according to published marker genes was performed on clusters at resolution 3.2. Highly similar clusters were merged. One cluster consisting of 616 cells, driven largely by low quality, was removed, resulting in 83 847 retained cells. As none of the assessed cluster resolutions accurately separated CD4 SP and CD8 SP cells, the CD4 SP and CD8 SP annotations were performed by re-integrating the SP thymocytes, re-doing dimensionality reduction, and re-clustering at resolution 1.2. The proportions of cells of each cell type from each donor are shown in **Supplementary Table S8**.

#### Prediction of cell-cell interactions with CellPhoneDB

Cell-to-cell signalling between annotated populations was inferred by CellPhoneDB v.2.0.0 (102, 103) using default parameters after random downsampling of the annotated dataset to 30 000 cells by use of the sample() function without replacement in base R.

#### Subclustering of thymocytes, DCs, B cells, and TECs

For more focused analyses, subsets of thymocytes, DCs, B cells, and TECs were prepared. For the thymocyte subset, thymocyte clusters from the full dataset were subsetted and re-integrated. Scaling and dimensionality reduction was performed with or without regressing out cell cycle scores, calculated according to expression of lists of cell cycle genes implemented in Seurat. Cell labels from the full dataset were retained. Pseudotime analysis was performed using Monocle3 v.1.0.0 (43, 104–107), with the root node placed in the DN(P) cluster.

The DC subset was prepared from DC populations stemming from APC-enriched and CD45-depleted samples. For integration by Seurat’s CCA-based integration anchor workflow, the k.weight parameter in the IntegrateData() function was reduced to 50 due to low number of cells for some samples. Dimensionality reduction was performed with 30 principal components kept for UMAP, and re-clustering was performed at resolution 0.4. This revealed the presence of clusters expressing T cell, B cell, and stromal cell markers, respectively, with a large proportion of events exhibiting high doublet scores. These clusters were therefore filtered out, and the retained cells were subjected to a new integration, dimensionality reduction and clustering. Manual annotation was performed for clusters at resolution 1.0, with merging of highly similar clusters.

The B cell subset was reanalysed in a similar manner as described for the DC subset, from the B cell and B plasma populations stemming from APC-enriched and CD45-depleted samples, but with one CD45-depleted sample excluded due to low number of cells. In contrast to the analysis of the DC subset, default setting was used for k.weight during integration, and cluster resolution was set to 0.6.

The TEC subset was prepared from TEC populations stemming from CD45-depleted samples. After re-running integration, dimensionality reduction with 30 principal components retained for UMAP, and clustering at resolution 0.8, manual annotation was performed, and highly similar clusters were merged. Two clusters were filtered out due to expression of genes related to mural and endothelial cells, respectively.

The TEC datasets published by Park et al. (38) and Bautista et al. (30) were reanalysed after subsetting on samples from young paediatric donors only, including one donor for the Park et al. TEC dataset, and two donors for the Bautista et al. TEC dataset. Integration was performed for the two donors from the Bautista et al. dataset by the CCA-based integration anchor workflow. For both datasets, UMAP was calculated using the first 30 principal components from PCA. These reanalysed reference datasets were then used for predicting annotations for our TEC subset by Seurat’s label transfer approach.

#### Differential expression (DE) analysis

Both in the full dataset and in each of the subsets, differentially expressed genes between clusters were calculated by the FindMarkers() function in Seurat, setting min.pct to 0.25 and only.pos to TRUE.

#### Analysis of PBMC data

For the PBMC data, ambient RNA correction, calculation of doublet scores, and filtering was performed as for the thymic samples, resulting in 19 651 retained cells. Then, Seurat’s SCTransform() function was applied before integration of all five PBMC samples by the CCA-based integration anchor approach, and 30 principal components from PCA were used for UMAP. Cell labels were predicted based on label transfer from a comprehensive PBMC CITE-seq dataset published by Hao et al. (66). Then, samples were reprocessed using the log normalised pipeline in the same manner as for thymic samples, except for retaining 30 principal components for UMAP, in order to facilitate mapping of the thymic samples onto the PBMC UMAP coordinates by Seurat’s MapQuery() function.

#### Analysis of spatial transcriptomics data

For the spatial transcriptomics data, consisting of 18 329 spots across eight tissue sections, normalisation was performed using SCTransform, and integration was performed by Seurat’s CCA-based integration anchor approach. Dimensionality reduction was performed first by PCA, and then on the first 30 principal components by UMAP. Clustering was performed by the Louvain algorithm using a range of resolutions from 0.2 to 2.0. Clusters at resolution 1.4 were annotated according to anatomical niche, inferred by placement of the spots making up the cluster on the H&E images and by differentially expressed genes calculated as for the single cell sequencing data.

In order to deconvolute individual spots into their cell type composition, SPOTlight v.0.1.4 (108) was used. The single cell RNA sequencing dataset was used as the reference, and marker genes for seeding the SPOTlight algorithm were calculated using the FindAllMarkers() function from Seurat, with min.pct and lofgc.threshold set to 0, and only.pos set to TRUE. As specific topic profiles could not be inferred for highly similar cell populations, similar clusters in the reference were merged prior to identifying differentially expressed genes. SPOTlight was then run using these differentially expressed genes together with the top 3000 highly variable genes after downsampling the reference dataset to 100 cells from each annotated population.

#### Figure preparation

Visualisations were prepared using Nebulosa v.1.2.0 (109) and ggplot2 v.3.3.5 (110), in addition to functions implemented in Seurat and SPOTlight. **Figure 1A** was created using content from 10x Genomics, and **Figures 1A**, **9** were created using content from Servier Medical Art.

## Data availability statement

The datasets presented in this study can be found in online repositories. The names of the repository/repositories and accession number(s) can be found below: GSE207206 (GEO).

## Author contributions

Conseptualisation, MH, SF, HH, TR, MS, and BL; Methodology, MH, SF, HH, MS, and BL; Formal analysis, MH; Investigation, MH, SF, HH, DT, MF, and MS; Resources, DT, MF, and K-AD; Data curation, MH, SF, HH, and BL; Writing – Original Draft, MH, SF, HH, and BL; Writing – Review & Editing, MH, SF, HH, DT, MF, K-AD, TR, XT, MS, and BL; Visualisation, MH, SF, HH, and BL; Supervision, TR, XT, MS, BL; Project Administration, BL; Funding acquisition, BL. All authors contributed to the article and approved the submitted version.

## Glossary

**Table:**

aDC	activated dendritic cell
ADT	antibody-derived tag
APC	Antigen-presenting cell
CCA	Canonical correlation analysis
CITE-seq	Cellular Indexing of Transcriptomes and Epitopes by sequencing
cTEC	Cortical thymic epithelial cell
DC	Dendritic cell
DN	Double negative
DP	Double positive
Endo	Endothelial cell
Fibro	Fibroblast
GC	Germinal centre
H&E	Hematoxylin and eosin
TCR	T cell receptor
MHC	Major histocompatibility complex
Mono	Monocyte
mTEC	Medullary thymic epithelial cell
NK	Natural killer
NMF	Negative matrix factorisation
PBMC	Peripheral blood mononuclear cell
PCA	Principal component analysis
pDC	plasmacytoid dendritic cell
RBC	Red blood cell
SP	Single positive
TCR	T cell receptor
TEC_(myo)_	Myeloid thymic epithelial cell
TEC_(neuro)_	Neuroendocrine thymic epithelial cell
T_reg_	Regulatory T cell
T_reg_(diff)	Differentiating T_reg_
UMAP	Uniform manifold approximation and projection
