# Supplementary material for: Multimodal human thymic profiling reveals trajectories and cellular milieu for T agonist selection
Source: Front Immunol. 2023 Jan 20;13:1092028. doi: 10.3389/fimmu.2022.1092028 (PMC9895842; doi:10.3389/fimmu.2022.1092028)
Supplement: Supplementary file 1 [file DataSheet_1.pdf]

Supplementary figures

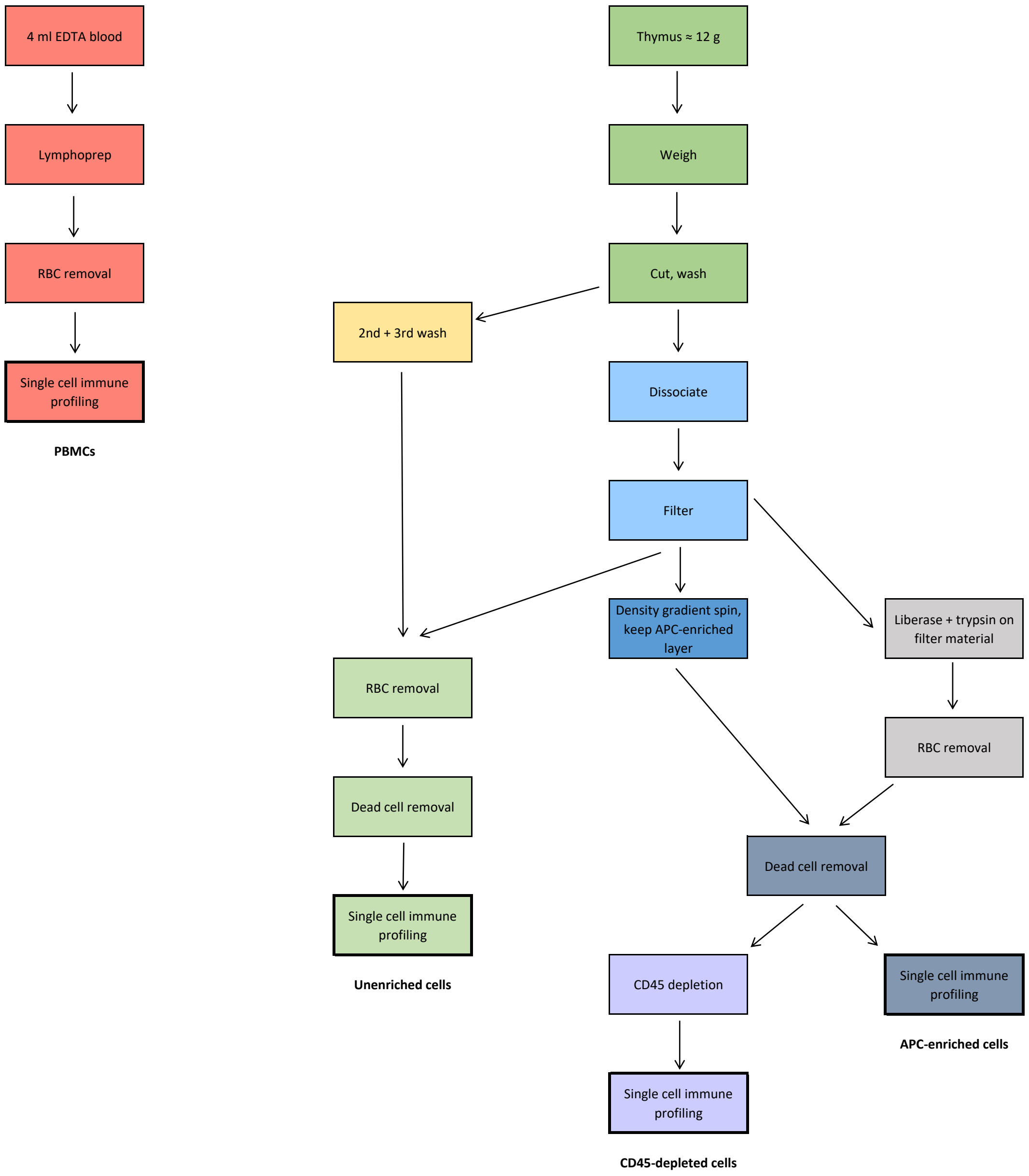

Supplementary Figure S1: Schematic overview of the protocol for dissociation and enrichment of thymic samples for single cell immune profiling. RBC = red blood cell.

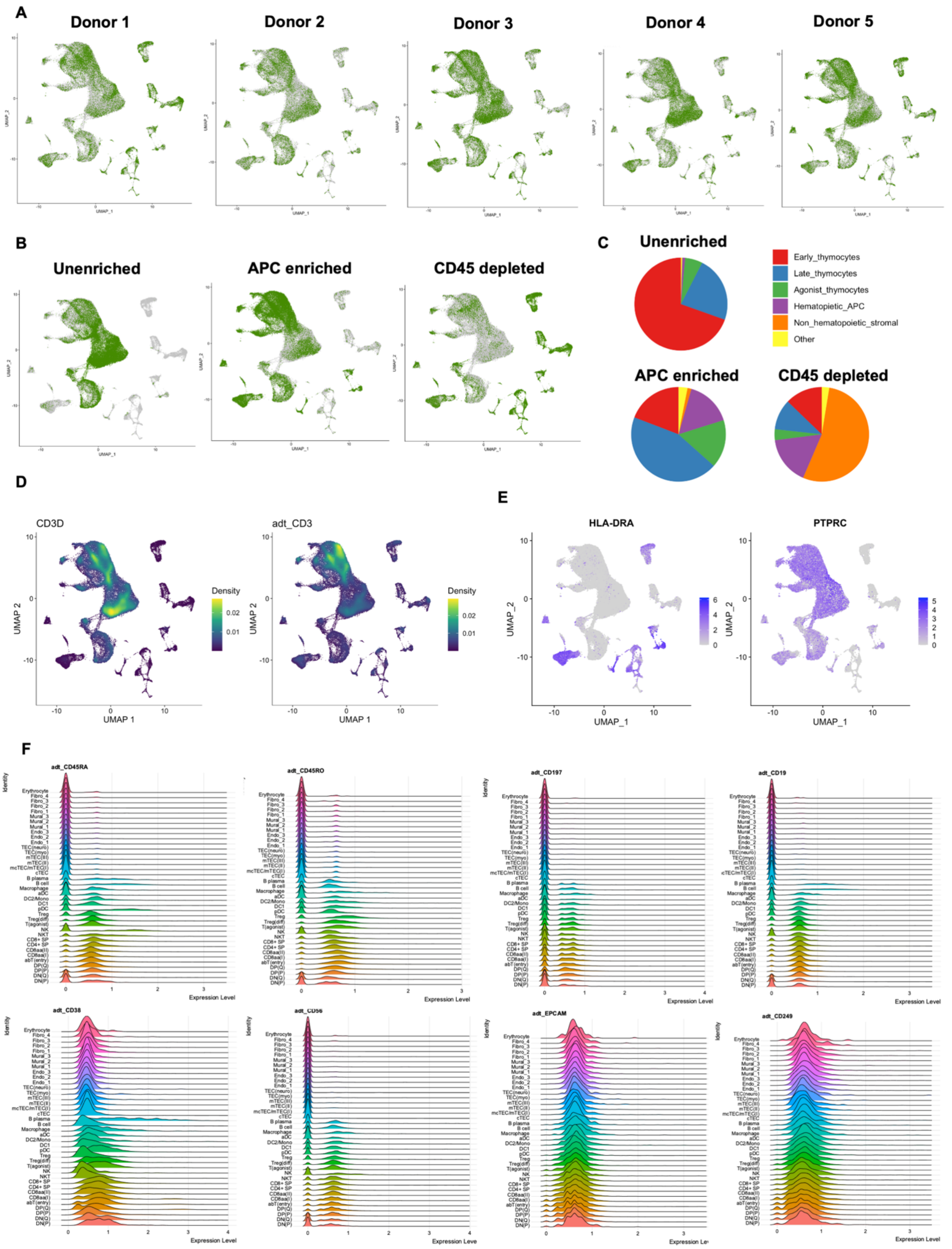

Supplementary Figure S2: Integrated CITE-seq dataset from thymic samples (n=15) at distinct stages of enrichment. A. Contribution of samples from each donor to the full, integrated thymic dataset. Events stemming from the respective donor indicated in green. **B.** Contribution of each enriched sample type (unenriched, APC-

enriched, or CD45-depleted) to the full, integrated thymic dataset. Events stemming from the respective sample type indicated in green. **C.** Pie charts indicating the distribution of cell types within each enriched sample type, grouped as “early thymocytes” (DN(P), DN(Q), DP(P), DP(Q)), “late thymocytes” ($\alpha\beta$T(entry), $CD4^{+}$ SP, $CD8^{+}$ SP), “agonist thymocytes” (CD8$\alpha\alpha$(I), CD8$\alpha\alpha$(II), $T_{(agonist)}$, T_{reg}(diff), T_{reg}), “hematopoietic APC” (pDC, DC1, DC2/Mono, aDC, B cell, B plasma), “non-hematopoietic stromal” (cTEC, mcTEC/mTEC(I), mTEC(II), mTEC(III), TEC(myo), TEC(neuro), Endo_1-3, Mural_1-3, Fibro_1-4), and “other” (NK, NKT, macrophage, erythrocyte). **D.** Density plot created by Nebulosa, showing expression of *CD3D*/CD3 at RNA (left) and protein (right) level. adt = antibody derived tag. **E.** RNA level expression of *HLA-DRA* and *PTPRC* in the full dataset. **F.** Expression of selected proteins in annotated cell populations by antibody capture.

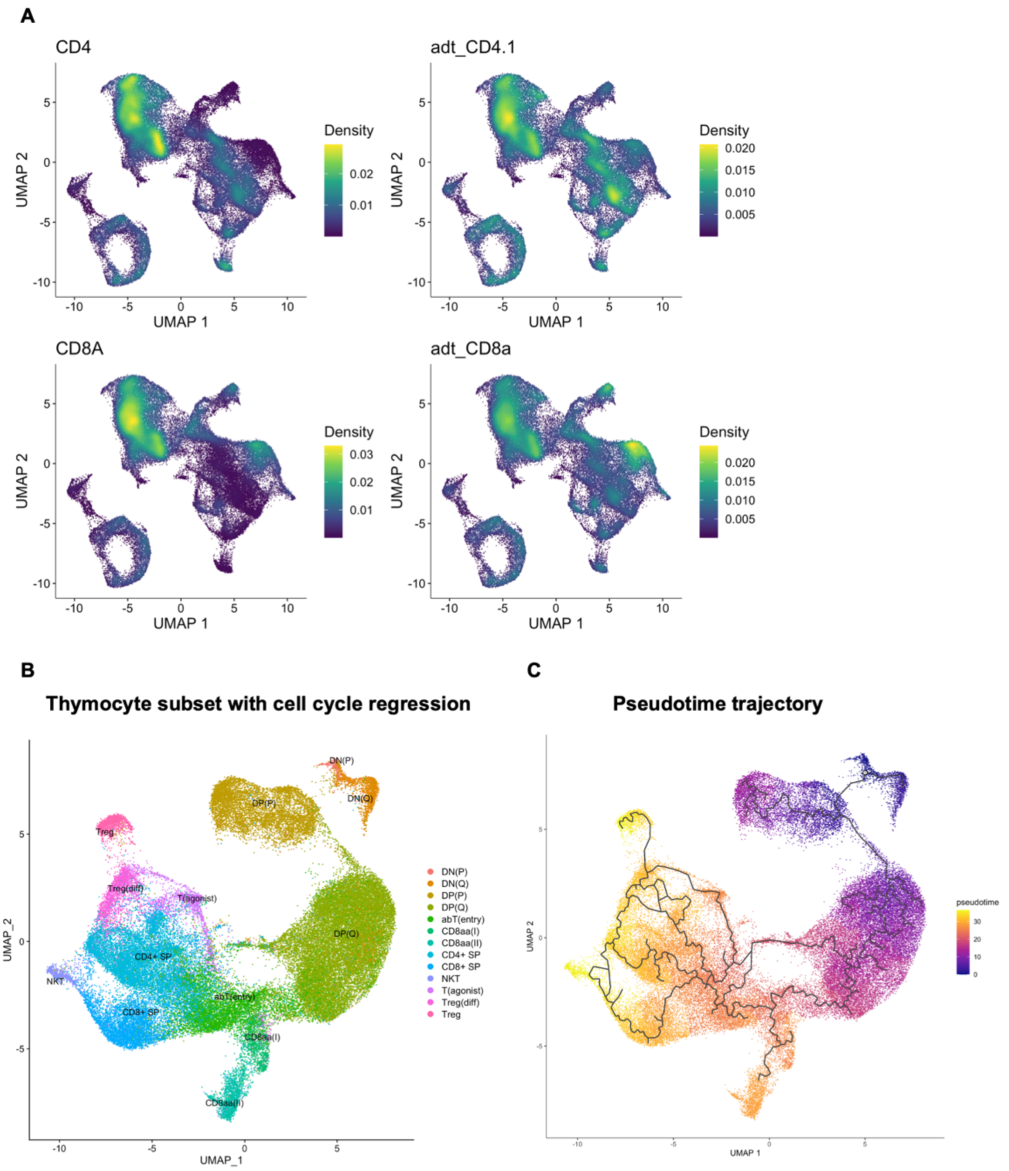

Supplementary Figure S3: Reanalysis of thymocyte subset. A. Density plot created by Nebulosa showing expression of CD4 and CD8A at RNA (left) and protein (right) level. adt = antibody derived tag. **B.** Annotated UMAP of thymocyte subset after regressing out the effect of cell cycle scores calculated according to gene lists provided by Seurat. **C.** Pseudotime analysis of thymocyte subset after cell cycle regression by Monocle3.

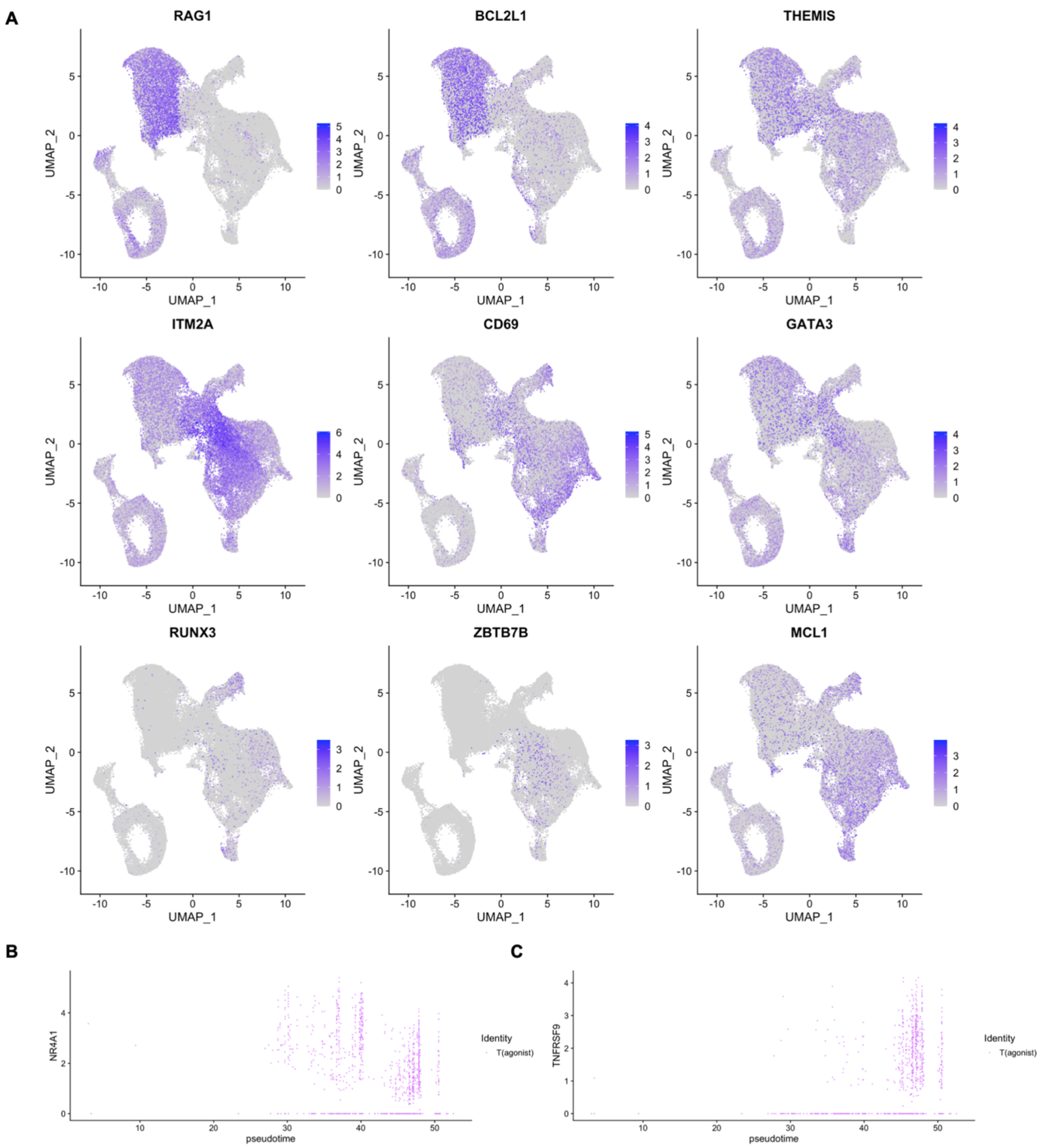

Supplementary Figure S4: Gene expression in the thymocyte subset. A. Expression of selected genes in thymocyte subset. **B.** and **C.** Expression of selected genes in the $T_{(agonist)}$ cluster along pseudotime.

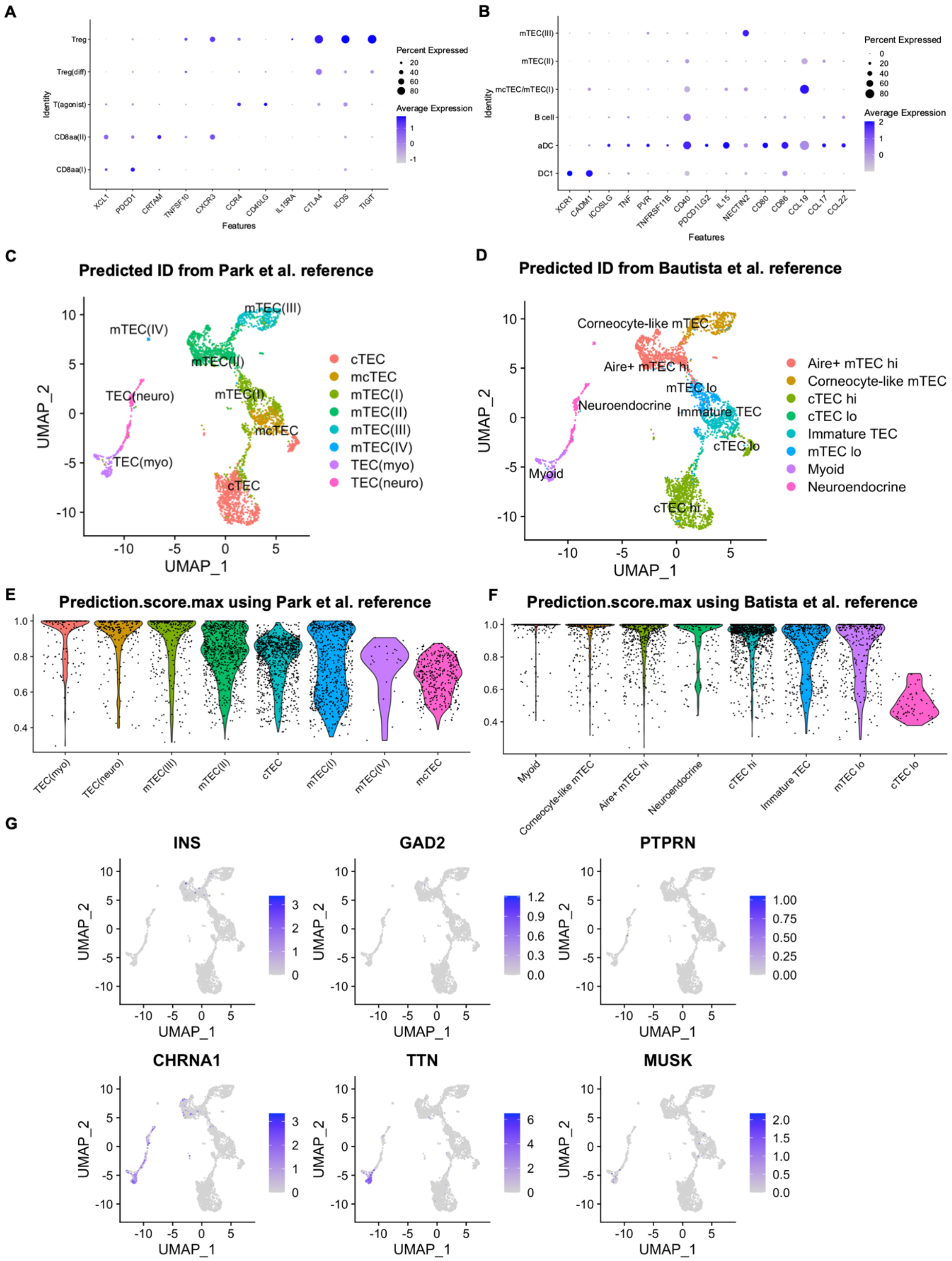
A
Treg
Treg(diff)
T(agonist)
CD8aa(II)
CD8aa(I)
Identity
XCL1
PDCD1
CRTAM
TNFSF10
CXCR3
CCR4
CD40LG
IL15RA
CTLA4
ICOS
TIGIT
Features
Percent Expressed
Average Expression
B
mTEC(III)
mTEC(II)
mcTEC/mTEC(I)
B cell
aDC
DC1
XCR1
CADM1
ICOSLG
TNF
PVR
TNFRSF11B
CD40
PDCD1LG2
IL15
NECTIN2
CD80
CD86
CCL19
CCL17
CCL22
C
Predicted ID from Park et al. reference
cTEC
mcTEC
mTEC(I)
mTEC(II)
mTEC(III)
mTEC(IV)
TEC(myo)
TEC(neuro)
UMAP_1
UMAP_2
D
Predicted ID from Bautista et al. reference
Aire+ mTEC hi
Corneocyte-like mTEC
cTEC hi
cTEC lo
Immature TEC
mTEC lo
Myoid
Neuroendocrine
E
Prediction.score.max using Park et al. reference
F
Prediction.score.max using Batista et al. reference
G
INS
GAD2
PTPRN
CHRNA1
TTN
MUSK

Supplementary Figure S5: Reanalysis of the epithelial cell subset and comparison to previously published datasets. A. and **B.** Expression of ligands/receptors across cell populations from the initial low-level annotations. **C.** and **D.** Predicted annotations for the TEC subset by label transfer using the Park et al. and Bautista et al. paediatric TEC subsets as reference. **E.** and **F.** Predictions scores for the label transfer from Park et al. and Bautista et al. paediatric TEC subsets. **G.** Expression of selected tissue restricted antigens in the TEC subset.

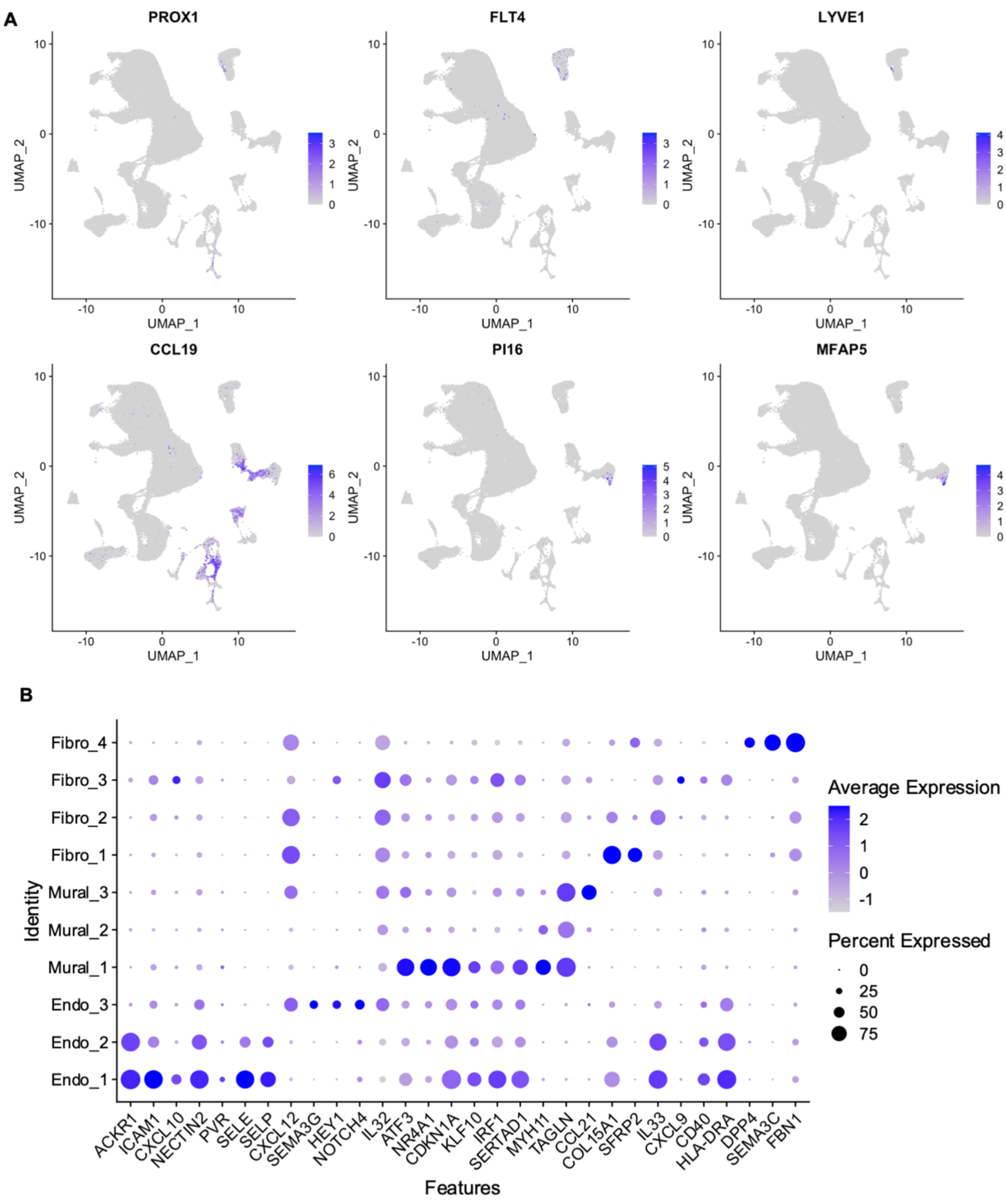

Supplementary Figure S6: Expression of selected genes associated with non-TEC stromal cells. A. Expression of selected genes associated with stromal cell populations in the full thymic dataset. **B.** Expression of selected genes across the non-TEC stromal cell populations.

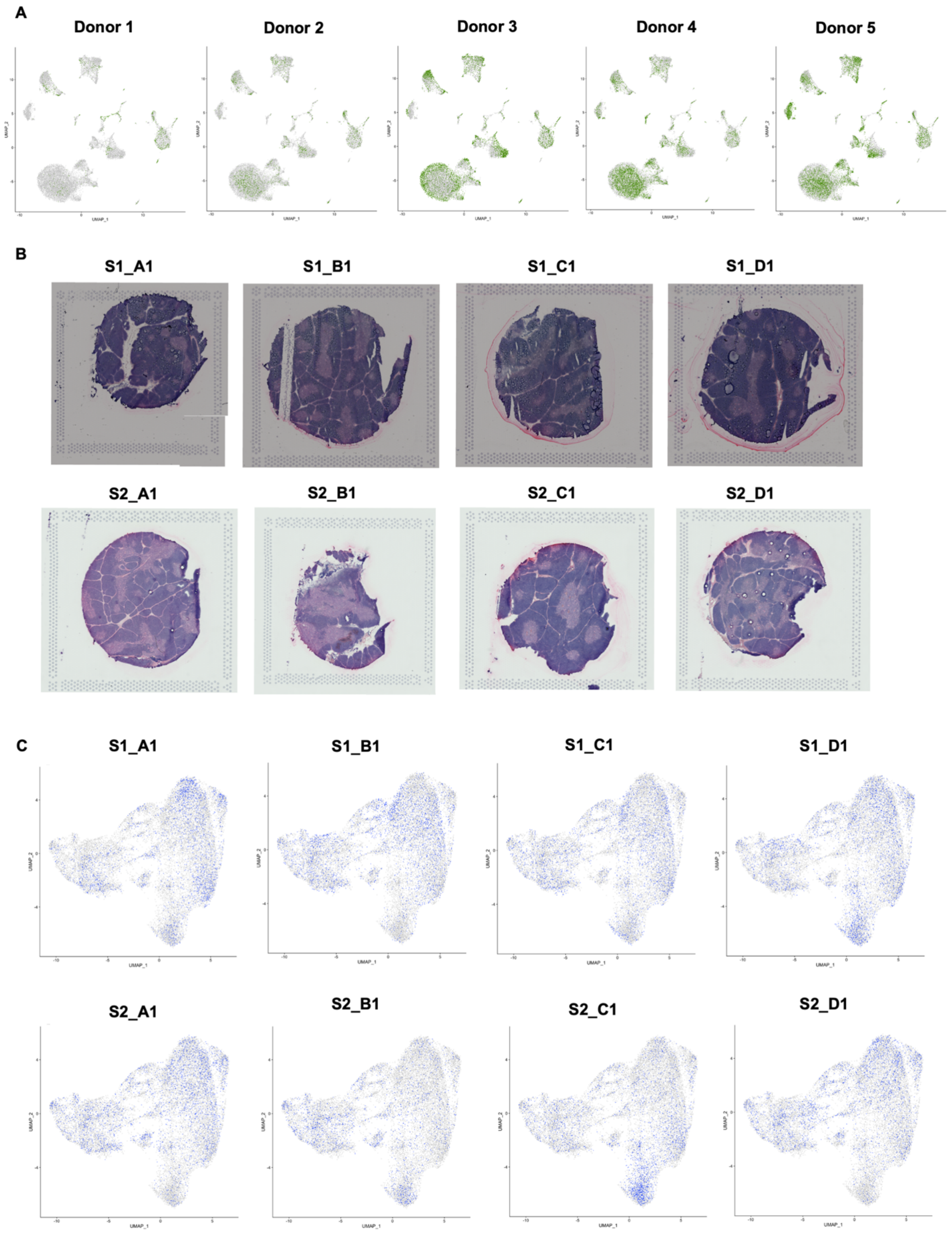
A
Donor 1
Donor 2
Donor 3
Donor 4
Donor 5
B
S1_A1
S1_B1
S1_C1
S1_D1
S2_A1
S2_B1
S2_C1
S2_D1
C
S1_A1
S1_B1
S1_C1
S1_D1
S2_A1
S2_B1
S2_C1
S2_D1

Supplementary Figure S7: Donor distribution across PBMC and spatial transcriptomics datasets. A. Contribution of samples from each donor to the integrated PBMC CITE-seq dataset. Events stemming from the respective donor indicated in green. **B.** H&E stained images of tissue sections included in the spatial transcriptomics dataset. **C.** Contribution of samples from each donor to the integrated spatial transcriptomics dataset. Events stemming from the respective donor indicated in blue.

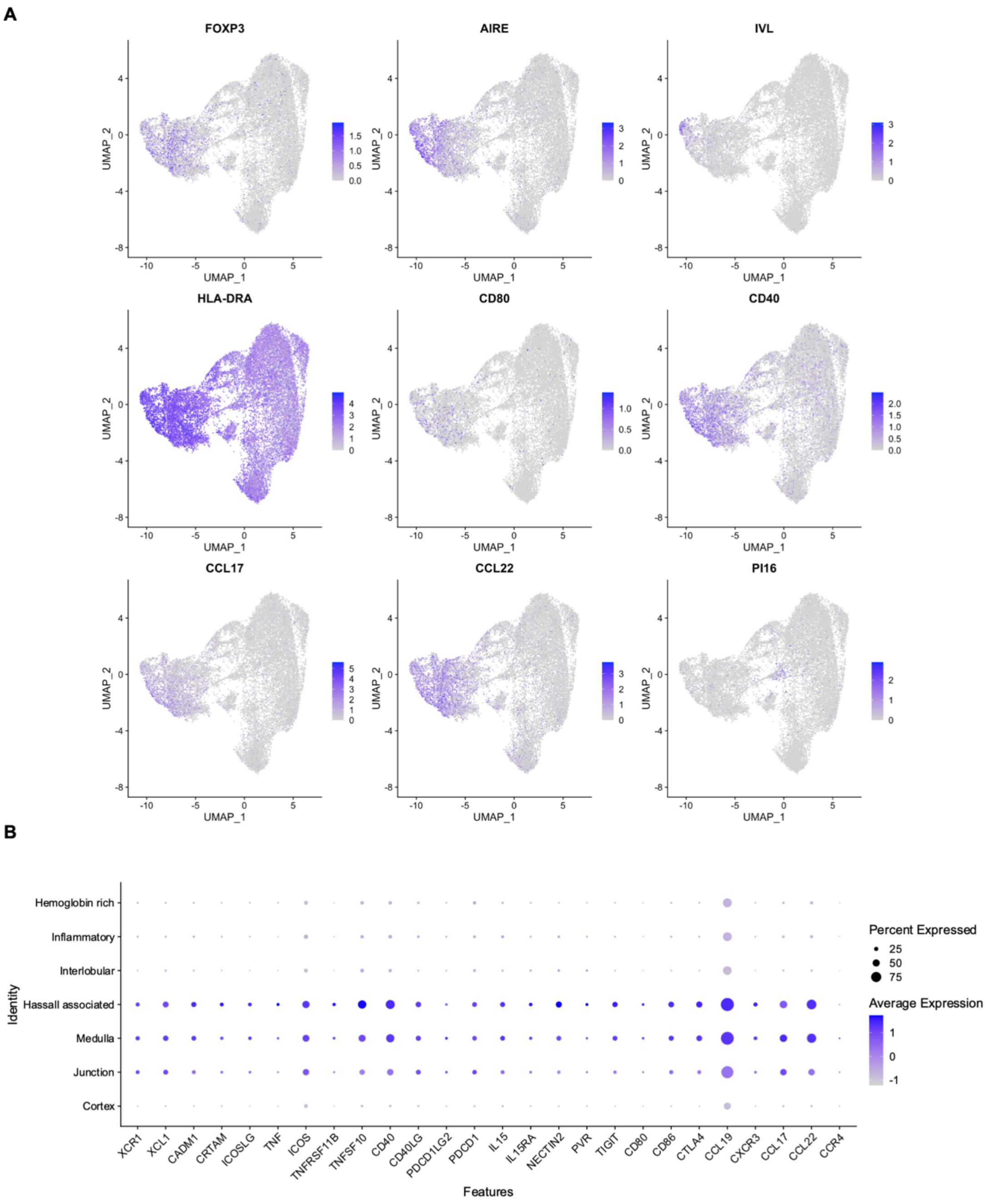

Supplementary Figure S8: Expression of selected genes in the spatial transcriptomics dataset.

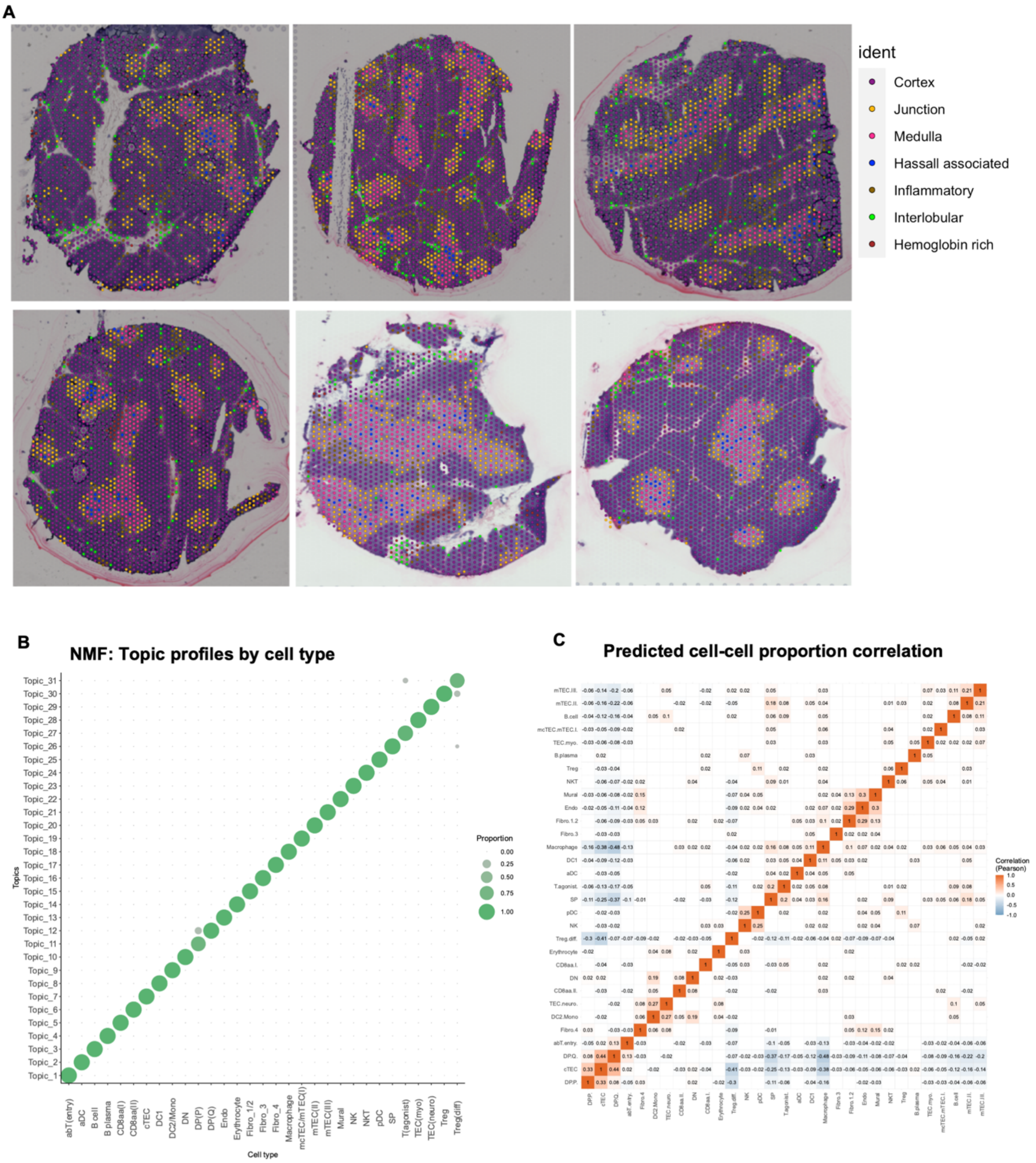

Supplementary Figure S9: Annotation and SPOTlight cell type deconvolution of spatial transcriptomics dataset. A. Tissue sections coloured according to annotations from Figure 6A. **B.** Proportion of cell type/state specific topic profiles by cell type/state. NMF = negative matrix factorization. **C.** Pearson correlation between predicted cell types/states.

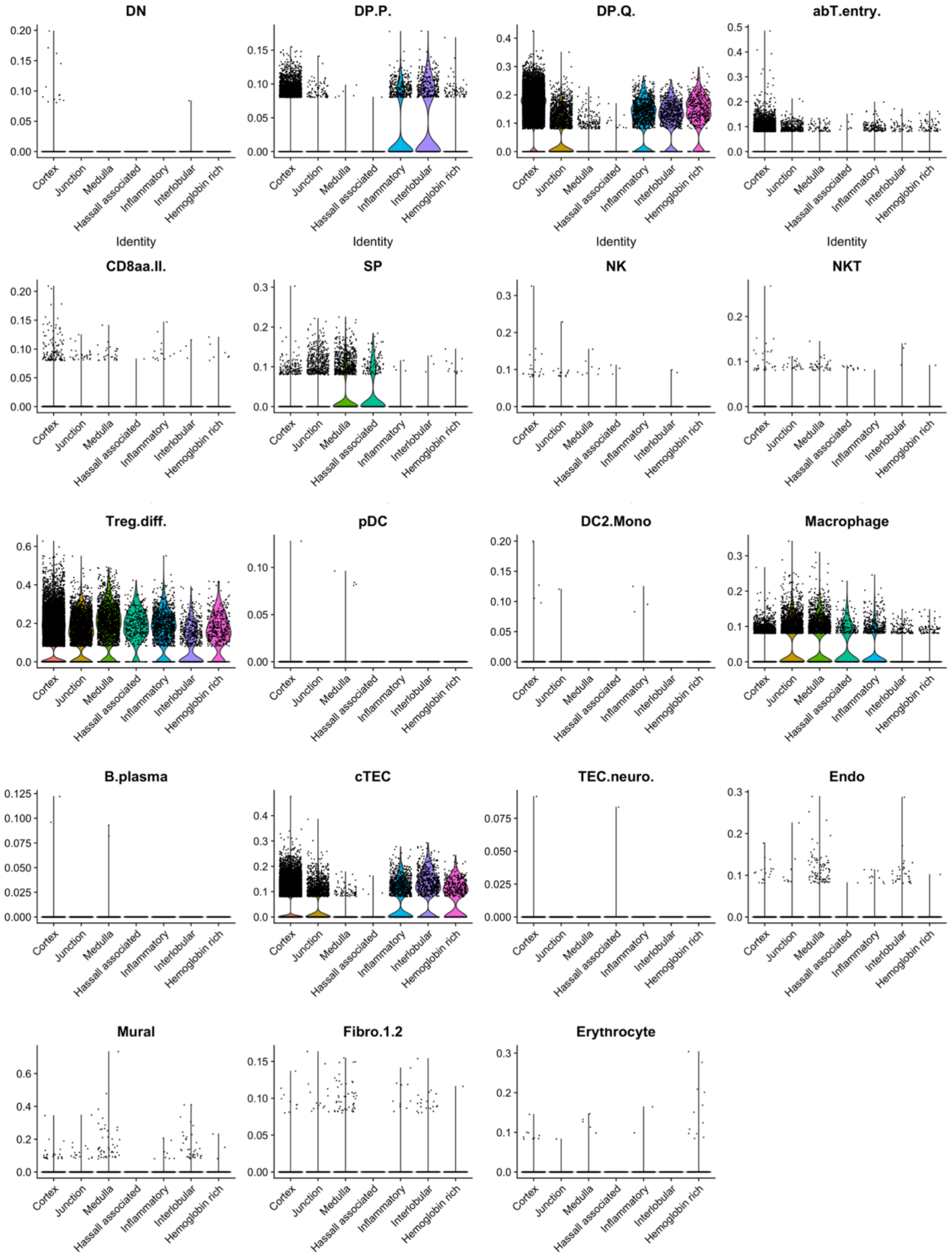

Supplementary Figure S10: Predicted proportion of selected populations among annotated tissue spots.

Supplementary tables

Supplementary Table S1: Donor metadata

Donor	Sex	Age	Application
D1	Male	7 days	Single cell immune profiling
D2	Male	2.5 months	Single cell immune profiling
D3	Female	9 months	Single cell immune profiling
D4	Female	1.6 months	Single cell immune profiling
D5	Male	13.5 months	Single cell immune profiling
S1_A1	Male	5 months	Spatial transcriptomics
S1_B1	Male	10 days	Spatial transcriptomics
S1_C1	Male	4 months	Spatial transcriptomics
S1_D1	Female	9 months	Spatial transcriptomics
S2_A1	Male	5.5 months	Spatial transcriptomics
S2_B1	Male	5 months	Spatial transcriptomics
S2_C1	Female	5.5 months	Spatial transcriptomics
S2_D1	Female	12 months	Spatial transcriptomics

Supplementary .csv table titles

Supplementary Table S2: Antibodies used for staining of CITE-seq samples

Supplementary Table S3: Differentially expressed genes for annotated clusters in the full dataset.

Supplementary Table S4: Differentially expressed genes for annotated clusters in the thymocyte subset.

Supplementary Table S5: Differentially expressed genes for annotated clusters in the DC subset, B cell subset, and TEC subset.

Supplementary Table S6: Differentially expressed genes for annotated clusters in the spatial transcriptomics dataset.

Supplementary Table S7: Quality control statistics for CITE-seq and spatial transcriptomics datasets.

Supplementary Table S8: Cell type proportions of annotated cell populations across samples.
